# Behavior Strategy Analysis Based on the Multi-Stakeholder Game under the Plastic Straw Ban in China

**DOI:** 10.3390/ijerph182312729

**Published:** 2021-12-02

**Authors:** Tinggui Chen, Yuling Zhang, Jianjun Yang, Guodong Cong, Guozhang Jiang, Gongfa Li

**Affiliations:** 1 School of Statistics and Mathematics, Zhejiang Gongshang University, Hangzhou 310018, China; ctgsimon@mail.zjgsu.edu.cn (T.C.); swagylll@hotmail.com (Y.Z.); 2 Academy of Zhejiang Culture Industry Innovation & Development, Zhejiang Gongshang University, Hangzhou 310018, China; 3 Department of Computer Science and Information Systems, University of North Georgia, Oakwood, GA 30566, USA; Jianjun.Yang@ung.edu; 4 School of Tourism and Urban-Rural Planning, Zhejiang Gongshang University, Hangzhou 310018, China; 5 Hubei Key Laboratory of Mechanical Transmission and Manufacturing Engineering, Wuhan University of Science and Technology, Wuhan 430081, China; whjgz@wust.edu.cn; 6 Key Laboratory of Metallurgical Equipment and Control Technology of Ministry of Education, Wuhan University of Science and Technology, Wuhan 430081, China; ligongfa@wust.edu.cn

**Keywords:** plastic straw ban, straw substitute, multi-stakeholder behavior, evolutionary game

## Abstract

Since 1 January 2021, China has banned nondegradable disposable straws in the catering industry. To promote the enforcement of the ban of plastic straws and improve the relationship between economic development and environmental protection, based on the evolutionary game method, this paper constructs the game model from the supply side and the demand side, respectively. Subsequently, through the dynamic equation, stable system evolution strategy is obtained. Furthermore, simulation is conducted to test the influence of the main parameters in the model on the evolution of system strategy. The results show that (1) the change of the government strategy mainly depends on its regulation costs and revenue, while the production strategy of a company is affected by the government and consumer strategies. (2) From the perspective of enterprise supply, government subsidies can promote technological innovation and develop new plastic straw substitutes. However, government penalties have little effect on violating enterprises. In addition, from the perspective of enterprise demand, with the collaboration of enterprises and consumers, it is easier for enterprises to carry out technological innovation. (3) Consumer acceptance of the substitutes for disposable plastic straws as well as online comments have a decisive influence on the enterprises’ selections for research and development (R&D) strategies.

## 1. Introduction

At present, plastics are widely used in various fields of our life, but their irregular production, utilization, and disposal cause waste of resources and pollution of the ecological environment and even affect public health. Moreover, plastics pose a great threat to the marine environment. According to statistical data from the United Nations, plastic fragments are found in oceans all over the world, and the observed concentration of plastic fragments per square kilometer of the sea surface is as high as 580,000 pieces [1]. In recent years, people have been pushing to eliminate the use of disposable plastics, especially the use of disposable plastic straws, in order to minimize the flow of plastics into the environment [2]. Correspondingly, governments and enterprises in various countries have taken relevant measures to deal with plastic pollution.

In 2014, the United Nations Environment Program called upon stopping marine plastic pollution. In response to this call, countries around the world have launched policies related to plastic pollution control. More than 40 countries have introduced measures to prohibit or levy taxes on the use of plastics. In addition, in the Commission work program for 2021, the revisions and initiatives linked to the European Green Deal climate actions, in particular, the climate target plan’s 55% net reduction target, are presented under the Fit for 55 packages. Plastic straws are one of the top 10 pollutants in the ocean. Many countries have also issued corresponding regulations. For example, in July 2018, Seattle became the first major city in the United States to ban plastic straws from restaurants. Then, Washington, D.C., banned straws in January 2019 and banned plastic straws altogether in July. In China, the State Council issued the “Notice on Restricting the Production and Sale of Plastic Shopping Bags” in 2008. Subsequently, in January 2020, the National Development and Reform Commission and the Ministry of Ecology and Environment in China issued the “Opinions on Further Strengthening Plastic Governance”, which required the prohibition of the production, sale, and use of nondegradable disposable plastic straws and would completely prohibit the use of nondegradable disposable plastic straws in the catering industry from 1 January 2021.

At present, the substitutes for traditional plastic straws are mainly paper straws and polylactic acid (PLA) straws, but not all companies can produce PLA straws. For companies that are unable to produce PLA straws, they still mainly produce paper straws. However, whether to change the production strategy is not finalized yet. Usually, the average production cost of traditional plastic straws is 0.03 yuan (CNY) per piece; paper straws are 0.08–0.09 yuan per piece, and PLA straws are 0.17–0.2 yuan per piece. Due to cost pressure, catering businesses in China mainly purchase paper straws to replace traditional plastic straws. Although the launch of disposable paper straws can serve the purpose of environmental protection, they are not favored by consumers. Some consumers think that “paper straws are prone to be soft”, “they have a paper smell”, “they cannot bite straws”, etc. These concerns reflect their reluctance to use paper straws. Generally, in the environmental protection revolution, consumers play the fundamental role, and their consumption behavior has an important impact on the environment, economy, and society [3]. At the same time, the production strategy of an enterprise mainly depends on the consumers’ needs and behaviors, and the government plays the role of supervising the production of the enterprise. Based on this, to reduce plastic pollution and protect the environment, it is necessary to study the relationship between the production strategy of straw companies, consumer use strategies, and government regulation strategies so as to provide a basis for the formulation and implementation of the company’s production plan.

At present, research on disposable plastic straw substitutes mainly focuses on the evaluation of plastic straw substitutes, covering the functionality [4], sustainability [5], and environmental impact of substitutes [6]. For example, Gutierrez et al. [4] found paper straws are not durable enough and typically cost more than their plastic counterparts. They lose their mechanical integrity once they have been in contact with a typical beverage, and some brands’ straws could change the taste of the drink. In fact, promoting the production and use of traditional plastic straw substitutes aims to resolve plastic pollution. Enterprises and consumers play important roles in promoting disposable plastic straw substitutes and environmental protection. However, such literature is short of a study on enterprise and consumer behavior strategies. In recent years, the problem of environmental pollution has become increasingly serious, and a large number of studies on how to improve environmental problems is arising, mainly testing the effectiveness of policy implementation through questionnaire surveys [7], lifecycle assessment (LCA) [8], and using the evolutionary game theory to resolve problems such as waste recycling construction [9], plastic waste recycling [10], and encouraging green production [11]. To effectively resolve the problem of plastic pollution, all stakeholders need to work together. Meanwhile, the long-term effectiveness of policy enforcement depends on balancing interests of the involved stakeholders [12].

The new version of the “plastic ban” emphasizes the prohibition of the production and use of disposable nondegradable plastic straws, prompting most enterprises to produce paper straws instead of traditional plastic straws. However, consumers are not willing to accept paper straws. Based on that, in the background of the ban on plastic straws, it is valuable to discuss how the production strategy of enterprises can meet the needs of environmental protection, consumers’ sense of experience and improve their own revenue at the same time and how the government can supervise and promote the innovation of production enterprises to improve the relationship between environmental protection and economic development. Generally, when consumers drink, a straw is a necessity. Therefore, this paper focuses on how to guide enterprises to carry out technological innovation, change production strategies, and promote them to produce plastic straw substitutes with good functionality and environmental protection.

This paper studies the plastic straw ban at first, then determines the crawling content and time range of online comment data based on the policy content and analyzes the emotion of comments. Based on the results of policy analysis and emotion analysis, the government–enterprise game model and the government–enterprise–consumer game model are constructed from the supply level and the demand level of straw production enterprises, respectively. Finally, these two models are verified through simulation. When constructing an evolutionary game, firstly, according to the problem background, the status of government, enterprises, and consumers, their expenses and benefits of each party’s strategies are assumed. Subsequently, the payment matrix and the replicator dynamic equation are constructed. At last, the stability strategy of the subject is analyzed by solving the differential equation. The government–enterprise game model is used to explore how the enterprise production strategy changes and how the government measures the effect of the enterprise production strategy when the enterprise only considers its own production capacity. At the same time, based on the government–enterprise model, the government–enterprise–consumer model introduces the factors of consumer acceptance and network comments on plastic straw substitutes and discusses the changes of the enterprise production strategy as well as the impact of government measures, consumer acceptance, and network comments on the production strategy when enterprises consider their own production capacity and consumer demands.

The structure of the paper is organized as follows: Section 2 is literature review, Section 3 introduces research framework of this paper, Section 4 introduces global regulations on plastic and analyzes the ban of plastic straws, Section 5 uses online review data to analyze consumers’ emotional tendencies towards the new “plastic ban” and plastic straw substitutes, Section 6 constructs two game models of a multi-stakeholder strategy and simulation analysis under the plastic straw ban, Section 7 is the results and discussions, and Section 8 is the conclusions.

## 2. Literature Review

In recent years, plastic pollution is becoming more and more serious. At the same time, it has also attracted attention of a large number of scholars to plastic pollution abatement. At present, plastic pollution is widespread in agricultural production [13], personal protective equipment [14], bottled water consumption [15], and other aspects of our life. In China, a series of environmental regulation policies to protect the environment have been formulated and enforced. Among them, the new “Plastic ban” policy promulgated in 2020 is used to resolve plastic pollution in the environmental regulation policy. This section mainly analyzes the literature from three aspects: plastic straw substitutes, the enforcement effect of the plastic ban, and the applications of the evolutionary game theory under the environmental regulation policy.

### 2.1. Study on the Substitutes of Disposable Plastic Straw

Regarding single-use plastic pollution, Liu et al. [16] examined Hanoi as a case study on single-use plastics used by households, and investigated the daily generation of single-use plastic waste through a survey. Plastic shopping bags were found to be the most prevalent single-use plastic used by households, followed by wrap/film, straws, coffee cups with plastic lids, cutlery, takeout containers, food packaging, party cups, bottles for water and beverages, and other items. In addition, from the political perspective, Clayton et al. [17] compared and analyzed single-use plastics policy measures in 13 English-speaking Caribbean countries. They found eleven countries had introduced legislative policies, with seven implementing fines and penalties for non compliance.They also found that successful policies involve multiple tools, including primary stakeholder engagement, sufficient lead time between policy announcement and implementation, and extensive public education campaigns. Considering college campuses in particular serve as hubs for single-use plastics, Bruchmann et al. [18] tested whether social comparison information could influence self-perceptions of single-use plastic consumption and motivate behavior change within the college campus environment. The results indicated that (relative to a non-comparison control), being above average at water bottle sustainability led students to be more satisfied with their sustainability efforts. The innovation of sustainable and environmentally friendly single-use plastic alternative materials and the joint participation of governments, enterprises, and the public were promising technologies and management approaches that could solve the problem of single-use plastic waste [19].Although plastic straws account for a small fraction of urban garbage, they are also found in marine and coastal waste, resulting in policies to curb or ban improper disposal from the political perspective. Neto et al. [20] aimed at surveying, categorizing, and analyzing the existing regulations on straw bans on the American continent (North, Central, and South America and the Caribbean). The result showed in Central America and the Caribbean, it is the regulations that are the primary tools providing environmental education. At present, there is relatively limited research on plastic straw substitutes. Scholars mainly evaluate the sustainability of substitutes and their potential pollution of the environment through LCA. Some of them also study the impact of plastic straw substitutes of different materials on consumers’ senses through experimental investigation. For instance, Beekman et al. [2] studied differences in the consumer perception of iced coffee beverages between plastic straws and alternative drinking conditions. Their outcomes showed that consumer experience and acceptability of iced coffee beverages could vary with drinking conditions. Chang and Tan [5] presented a comparative sustainability study of drinking straws via LCA. Furthermore, an analytic hierarchy process (AHP) was incorporated to aid decision-making on sustainability performance based on the triple bottom line of sustainable development covering environmental, economic, and social pillars. It was concluded that plastic straws are more sustainable than stainless steel straws. Boonniteewanich et al. [6] compared the carbon footprint between bioplastic straws and PP straws through the lifecycle. The results showed that the carbon footprint of bioplastic straws is higher than that of PP straws because of more waste from manufacturing. Jonsson et al. [21] tested consumer sensory properties and the durability of straws when soaked in water. The result showed paper, wheat, pasta, and rice straws all rated low on mouth feel and flavor liking, with off-flavors commonly reported in beverages consumed with these straws. Furthermore, plastic straws provided a superior sensory experience compared to other options. Chitaka et al. [22] compared the environmental impacts associated with five straw material options: disposable options (polypropylene, paper, and polylactide) and reusable straws (glass and steel). The assessment was conducted using the recipe midpoint (H) method to explore the potential marine pollution. In that paper, straws were found to have lower climate change emissions than plastic. In the study of single-use plastic pollution, the current situation and causes of plastic pollution are mainly studied. However, single-use straws are widely used in daily life, and the environmental harm caused by single-use straws is usually ignored. Therefore, the most sustainable and effective solution to the problem of single-use plastic straws is to develop less polluting alternatives. In the above research, scholars mainly analyzed sustainability, functionality, and impact on the environment of plastic straw substitutes. The results show that plastic straws have a good functionality, but pose a greater threat to the environment than the alternatives such as paper straws and biodegradable plastic straws. However, in the current research, the scholars only studied the experience and acceptance of using plastic straw substitutes, but did not analyze the impact of consumer acceptance of alternative products on the income of production enterprises from the perspective of the market environment nor considered the role of online comments on plastic straw substitutes on the income of production enterprises.

### 2.2. Research on the Effect of Plastic Ban Policy

With regard to the research on the effect of plastic ban policy, Wen et al. [8] quantified the environmental impacts of changes in the flow pattern and treatment methods of six types of plastic waste in 18 countries subsequent to the ban by the LCA method. The result indicated the ban could effectively improve the environment. Macintosh et al. [23] studied effectiveness and durability that a ban on single-use plastic bags introduced in the Australian Capital Territory. The results suggested the ban had not been overly effective in reducing plastic bag consumption. In addition, through the comparative business power of plastic industries, Behuria [24] explained whether bans of plastic bags in different countries were obstructed. The results indicated it did not satisfactorily explain varied implementations. However, countries that pursued services-based development strategies, which prioritized externally dependent sectors like tourism, were more likely to implement plastic bag bans. Besides, using a questionnaire survey, Omondi and Asari [25] explored consumer attitude towards the ban and reusable bag usage behavior. The results indicated that the ban had favorable support of about 67% from consumers. However, the perceptions towards cleanliness and waste management were different between urban and rural respondents, which highlighted differences in awareness creation and enforcement of the ban. Bharadwaj et al. [26] investigated the behavioral response of consumers and retailers to the plastic bag ban policy in different municipalities of Nepal. They investigated the use by both consumers and retailers of single-use versus reusable plastic bags and estimated different types of plastic bags using ordinary least squares. The results suggested that the perceived sanction was a critical determinant of plastic bag use. Herberz et al. [27] investigated whether banning single-use plastic items was an appropriate strategy to protect the environment. Product lifecycle assessment was conducted for single-useplastic and single-use non-plastic alternatives. The lifecycle impacts of the two product categories were compared and scaled according to the European Union (EU) consumption of 2016. The results showed that a single-use plastic ban would decrease plastic marine pollution in the EU by 5.5% which equated to a 0.06% decrease globally. With an in-depth investigation of plastic bag policies through the lens of diffusion research, Knoblauch et al. [28] found that industrialized countries had mostly adopted plastic bag taxes, while developing countries had mainly introduced plastic bag bans. The above scholars studied the enforcement effect of the plastic ban policy from the aspects of the lifecycle of plastic goods, commercial strength of the plastic industry, and the public’s perception of the policy. The results show that the enforcement effects of plastic ban policies in different countries are different. However, only few consider that the policy involves the behavior decision-making of stakeholders, and whether the policy enforcement effect is effective in the long term depends on whether the interests of stakeholders can be balanced [12].

### 2.3. Applications of the Evolutionary Game Theory under the Environmental Regulation Policy

At present, the game theory is widely used in evaluating environmental rules, regulations, and policies. It combines the participants’ interests of environmental sustainability. For instance, to study the behavioral decision-making of stakeholders in construction and demolition (CDW) recycling under environmental regulations, Shen et al. [9] considered the limited rationality of stakeholders, and an evolutionary game model including contractors and manufacturers of construction materials was proposed based on the prospect theory of behavioral economics. The results indicated that only when the perceived benefits of one or both stakeholders for participation under the environmental regulation exceeded those for non-participation could the CDW recycling system eventually evolve to a stable state in which both stakeholders would choose to participate. Wang et al. [10] applied the dynamic evolutionary game theory and included government as a player in order to shed light on the impact of government interventions on the participation of other stakeholders in the recycling and reuse of plastic waste. Simulation results indicated incentives or penalties of the government increased the probability that stakeholders would participate in the recycling process. Furthermore, to explore the relationships between the green activity strategy of the supply chain and the supervision behavior decision of governments and to investigate the effect of environmental regulations, including supervision and the reward and punishment mechanism, on the green activity strategies of suppliers and manufacturers, Xu et al. [11] established a three-population model of suppliers, manufacturers, and governments based on the evolutionary game theory and analyzed the evolutionary stability strategies (ESS) of their unilateral and joint behaviors. The results indicated the proportion of green suppliers and manufacturers in their groups determined whether the government implemented regulations and whether government regulations could incentivize corporations to adopt green behavior. Sheng et al. [29] conducted a theoretical analysis of the evolutionary stability strategies of the national government, local governments, and enterprises in China to explore the factors that influenced the strategies of various stakeholders by using a tripartite evolutionary game model. Numerical simulations were introduced to examine the asymptotic stability of various evolutionary stabilization strategies and the effects of parameter variation of these strategies. The results demonstrated that national government supervision was critical to achieving the goals of environmental regulation policies since supervision costs could influence the final evolutionary stability strategy. Through analysis of the evolutionary game in e-waste recycling industry in China, Wang et al. [30] focused on multiple evolutionary stability strategies of the game model corresponding to different stages of industry development. The results showed that the government should play a leading role in the development of the e-waste recycling industry. In addition, based on the conflict of interest between the government and shipping companies, Jiang et al. [31] constructed an evolutionary game model to analyze and test the dynamic changes of the participants’ decision-making. The outcomes demonstrated that, to inspire shipping companies to comply with the Emission Control Areas (ECA) regulations, the government should apply a strategy following dynamic penalties to make shipping companies more willing to execute the ECA regulation within less time. According to the problem of the amount of nondegradable wastes such as packing boxes and plastic bags increasing day by day, Wang et al. [32] established an evolutionary game model to analyze the effects of different decision-making behaviors of stakeholders on the construction of the recycling industry chain. The result showed that in the case of government intervention, the evolution results of the game system would always reach the ideal state, and the evolution speed is more greatly increased compared with the situation without government intervention. In the previous studies, the research methods of behavioral decision-making were mainly based on the evolutionary game theory. Moreover, some scholars use other methods to study behavioral decision-making. For example, Foschi et al. [33] presented the case of a small–medium enterprise and implemented a decision-making process to rethink the design of frozen food packaging in accordance with systemic and lifecycle thinking. The result showed that the solution provided a strong contribution to the reduction in the consumption of plastics and the prevention of marine pollution. Lewis et al. [34] assessed the influence of plastic bag waste on the environment by LCA, and the LCA results suggested that replacing one type of single-use bags (plastic) with another (e.g., paper or biodegradable plastic) might increase rather than decrease environmental impacts. Arı and Yılmaz [35] examined consumer attitudes and behaviors on the use of plastic and cloth bags and then proposed a structural equation model. Environmental consciousness regarding the use of plastic bags, social pressure, support for the banning of plastic bags, the intention to use cloth bags and behavior to reduce plastic bag use were employed as latent variables in the model. The result showed consumers who were environmentally conscious and felt under social pressure, tended to reduce the use of plastic bags and switch to using cloth bags. The aforementioned research on the applications of the evolutionary game theory under the environmental regulation policy involves the study on behavioral decision-making in construction waste recycling, plastic waste recycling, and e-waste recycling. Furthermore, the above scholars think that under the government intervention, the strategies of stakeholders can reach the ideal state in time and effectively, but there isa lack of research on behavioral decision-making in the field of plastic production.

To sum up, on the one hand, for the research on disposable plastic straw substitutes, scholars mainly study the sustainability and functionality of straw substitutes from the perspective of product materials or consumers, but neither considering the impact of comments on production revenue, nor studying the production decision and consumption behavior. On the other hand, evolutionary game theory has been widely used in the study of stakeholder behavior decision-making under the environmental regulation policy, but the above literature lacks the behavior decision-making problem of considering plastic production and utilization at the same time. Further, it does not consider the factors of consumer acceptance of substitutes, which will affect the consumers’ consumption behavior and enterprise production decision-making. Besides, the effect of policy enforcement depends on the cooperation of stakeholders. This needs to meet the interests of all parties and achieve balance in order to effectively promote policy enforcement. Based on the existing research, firstly, this paper takes the government, enterprises, and consumers as the stakeholders of the straw ban policy, constructs the government–enterprise evolutionary game model, and explores how the enterprise production strategy changes and how government measures affect the enterprise production strategy when the enterprise only considers its own production capacity. Secondly, this paper analyzes the emotional tendency of consumers after using paper straws through network comments and then takes it as an important factor to measure consumer acceptance. Subsequently, consumer acceptance andonline comments as the key influencing factors are introduced and the corresponding government–enterprise–consumer model is constructed so as to explore the internal game mechanism of the main strategy under the influence of these two factors.

## 3. Research Framework

**Figure 1:**
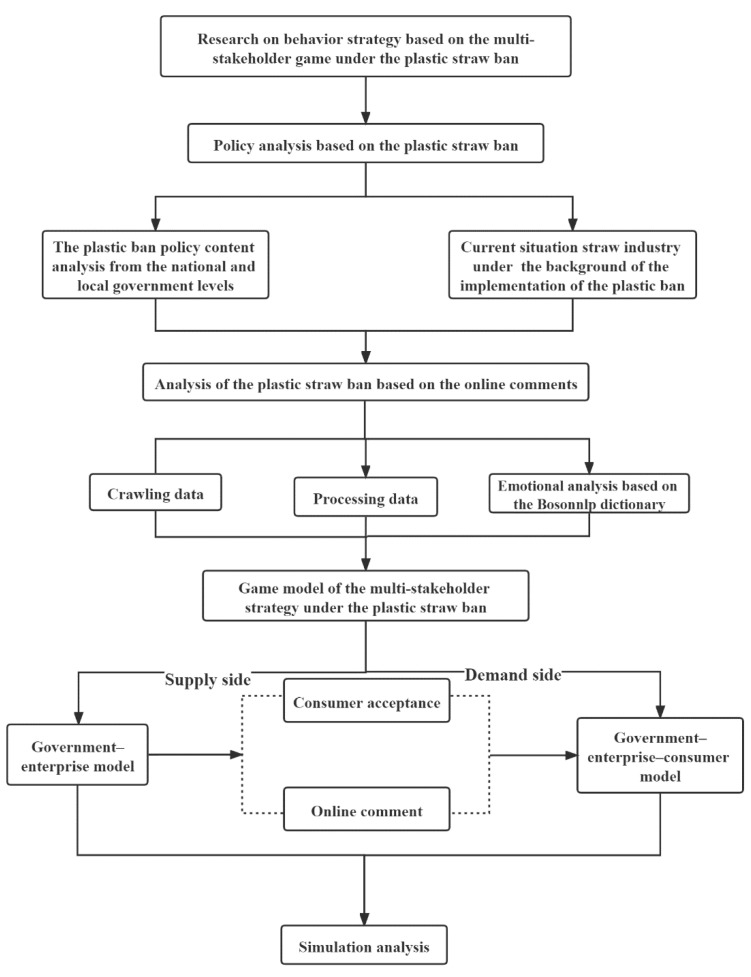
Research framework of this paper.

Firstly, this paper analyzes the content of the new “plastic straw ban” policy document from the national and local government levels in China and briefly describes the current situation of the straw industry in the background of the enforcement of the plastic straw ban policy. The policy analysis aims to determine the collection scope of online comments according to the theme of the policy text. In the context of the enforcement of the plastic ban, a brief description of the current straw industry can reflect the enforcement effect of the plastic restriction policy and analyze the resistance encountered in the process of policy enforcement. Then, according to the target task and time node requirements of the new version of the “Plastic ban”, the python crawler technology is used to crawl the comment data on Weibo from 19 January 2020 to 31 December 2020 and from 1 January 2021 to 30 April 2021. After the comment data have been cleaned, the emotion analysis is carried out based on the Bosonnlp dictionary. It is worth paying attention that the data from 19 January 2020 to 31 December 2020 were taken before the full enforcement of the policy, while the data from 1 January 2021 to 30 April 2021 were taken after the full enforcement of the policy. The purpose of emotion analysis of the two blocks of data is to reflect the public’s attitude towards the new “plastic ban” and the public’s attitude towards disposable plastic straw substitutes. Based on the previous analysis results, this paper takes straw manufacturing enterprises as the research object. First, from the enterprise supply level, this paper constructs the government–enterprise game model. After that, consumer acceptance and emotional tendency of online comments are introduced, and a government–enterprise–consumer game model from the enterprise demand level is constructed in order to explore the changes of the enterprise production strategy. Finally, the game model is analyzed by means of the simulation technology. The research framework of this paper is shown in Figure 1.

## 4. Policy Analysis

Before studying the behavior decision-making of policy stakeholders, we need to simply sort out the policies, analyze their content, and mine the relevant online comment data according to the theme in the policy text for the construction of a subsequent multi-stakeholder game model. Firstly, this section briefly describes the plastic policy at the international level. Then, it focuses on the analysis of China’s plastic policy and mines online comment information based on its policy theme. Finally, it introduces the current situation of the plastic straw market in the context of China’s restrictions on plastics.

### 4.1. Global Regulations of Plastics

In response to the growing production of plastic waste, the Basel protocol was amended in 2019 to control the export of plastic waste and took effect on 1 January 2021. Among the amendments adopted, waste minimization is stipulated. The United Nations Environment Program highlighted the Government of Canada which is consulting the public on future plastic straw regulations and the European Parliament and Council Directive aiming to implement similar measures. The Republic of Mauritius (Africa) banned several single-use plastics in early 2021, including plastic straws [36]. At the regional level, thirteen countries on the American continent enacted national regulations governing straws, including Antigua and Barbuda, the Bahamas, Barbados, Belize, Colombia, Costa Rica, Dominica, Grenada, Guatemala, Jamaica, Panama, Peru, and Santa Lucia [20]. Seattle (USA), a city that has historically had regulations governing the use of plastic packaging, initially excluding straws and single-use plastic cutlery, implemented a plastic straw ban in early July 2018. More recently, the United Nations Environment Assembly of UNEP gathered in Nairobi (Kenya) and passed a draft resolution on marine litter and microplastics. This draft document urges “all countries and other stakeholders to make responsible use of plastic while endeavoring to reduce unnecessary plastic use, and to promote research and application of environmentally sound alternatives [37].” On 14 July this year, the European Commission passed a crucial milestone by adopting the EU “Fit for 55” package to transform the European economy. It includes a major overhaul of the Emissions Trading System (ETS) to extend carbon pricing to shipping, aviation, transport, and buildings, accelerating the development of the renewable energy sector. It introduces a globally unprecedented carbon border adjustment mechanism (CBAM) for pricing imported carbon.

### 4.2. Analysis of Plastic Restriction Policies of the National and Local Governments in China

In 2001, China issued the “Emergency Notice on Immediately Stopping the Production of Disposable Foamed Plastic Tableware”. In 2008, the “Notice on Restricting the Production and Sales of Plastic Shopping Bags” was released, which was the first edition of the plastic ban. In January 2020, the “Opinion on Further Strengthening Governance” was published, i.e., the new version of the Plastic Restriction Order, which also included disposable nondegradable plastic straws into the scope of “plastic restriction” for the first time. In July that year, China issued the “Notice on Solidly Promoting the Control of Plastic Pollution” and published detailed standards for plastic products. In response to the national call, the provincial and municipal governments in China have also successively issued specific implementation measures. Governments at all levels formulate and issue policy documents on the basis of the “Opinions on Further Strengthening Plastic Pollution Control” and in combination with the actual situation in the province and the city, as shown in Table 1.

It can be seen in Table 1 that on the national level, phased goals for plastic pollution control are formulated, which require that the usage of nondegradable disposable plastic straws in the catering industry throughout the country be prohibited by the end of 2020 in China. Thereafter, governments at all levels in China have also formulated plans based on it. As a result, it is an urgent task for them to prohibit or restrict the production and utilization of disposable nondegradable plastic straws. Based on this, this paper focuses on the current prohibition goal for plastic straws.

### 4.3. Current Situation in the Straw Industry

The above plastic ban documents clearly require that the usage of nondegradable disposable plastic straws in the catering industry throughout the country be prohibited by the end of 2020. This is because straws are a common product in the catering industry and have enjoyed huge consumption for a long time. Due to a straw’s small size, its harmfulness had not attracted extensive attention of the society before. Nowadays, considering the serious environmental pollution caused by disposable plastic straws, China included it in the scope of the “plastic ban”, but it also brings challenges and opportunities to the straw industry.

On 1 January 2021, the plastic straw ban was enforced nationwide. In response to the national call, restaurants began using degradable straws and promoted the transformation of straw manufacturers. According to Tianyan survey data, more than 2000 enterprises nationwide are engaged in straw-related products. The enforcement of the new “plastic ban” has prompted enterprises with economic strength and production capacity to move from traditional plastic straws to paper straws, PLA straws, and other degradable straws. Here, we take Shuangtong Daily Necessities Co., Ltd. in Yiwu, the largest straw manufacturer in China as an example. Till 2018, the company put half of its production capacity into the production of degradable straws. In 2018, plastic straws were banned in Great Britain, the European Union, and some cities in the United States. Meanwhile, the export of degradable plastic straws by Shuangtong accounted for 70% of China’s exports and was restricted by COVID-19 in 2020. However, the demand for biodegradable straws increased further in January 2020 after the new “plastic ban” was introduced. In fact, due to the different materials and production technologies of degradable straws and plastic straws, the production cost of straws is also different, and the attitude of the end market towards alternative products is not clear, so some small and medium-sized enterprises have difficulties completing the technological transformation and their production strategies are still in the process of game.

## 5. Analysis of the Plastic Straw Ban Based on Online Comments

Generally, through analyzing the Weibo comment data, we can understand the feedback of the majority of Internet users on the enforcement of a policy. Here, the emotional value obtained from the emotion analysis of the online comment data is used to measure the impact of paper products on enterprises in the subsequent game evolution model which aims to explore the impact of comments on the production and R&D of new products (such as PLA straws, PP straws). Considering that 1 January 2021 was the first day that the plastic restriction ban was enforced nationwide, this paper uses the comment data before that time (19 January 2020–31 December 2020) to reflect the netizens’ attitude towards the new “plastic ban”. In addition, the comment data after that time (1 January 2021–30 April 2021) are used to reflect the social response after the full enforcement of the policy, which mainly involves the attitude of netizens towards plastic straw substitutes.

### 5.1. Data Selection and Pretreatment

Python crawler technology was used to crawl the relevant Weibo comments from 19 January 2020 to 30 April 2021, and the time of full enforcement of the “plastic ban” was taken as the demarcation timepoint. The total amount of Weibo comments before and after the full enforcement of the ban was 61,687, including 15,363 Weibo comments before the enforcement of the ban and 46,324 after the full enforcement of the ban.

Firstly, the comments were preprocessed, including (1) deletion of invalid comments that only contained numbers, special characters, @ user name or empty comments; (2) deletion of emoji expressions, special characters, punctuation marks, etc. from the comment text; (3) removal of comments unrelated to the study object.

The filtered comments are shown in Table 2: 11,744 comments under the topic “plastic straws will be banned by the end of the year”, accounting for 82.08% of all the comments before the ban was fully enforced;19,585 comments under the topic “consumers complain about paper straws for milk tea” and 11,295 comments under the topic “KFC uses wooden spoons”, accounting for 47.6% and 27.45%, respectively, of the comments after the policy was fully enforced.

### 5.2. Emotional Analysis

This section analyzes the emotion of the cleaned comment data based on semantic dictionaries. These dictionaries used here include the Bosonnlp emotion dictionary, the degree adverb dictionary, the negative words dictionary, the stop words dictionary, and the user-defined dictionary. In order to improve the accuracy of word segmentation, the user-defined dictionary contained new words that are not in the Jieba thesaurus, mainly including the words related to the research content and appearing frequently, e.g., “degradable straw”, “paper straw”, and “PLA straw”. The specific emotional analysis steps were as follows:

(1) Each comment was segmented according to the Jieba word segmentation library and the user-defined dictionary, and the segmented results were matched with the words in the stop word dictionary. If it existed in the stop word dictionary, it was eliminated, otherwise it was retained.
(2) Calculation of emotional values. Firstly, the word segmentation result was matched with the words in the Bosonnlp emotion dictionary, and the emotional value was retained; then, we looked for degree adverbs and negatives before emotional words. If present, we retained the degree value of degree adverbs and the number of negatives as the weight of emotion words; finally, the weighted summation method was used to calculate the emotional value of the comment data.
(3) Determination of the emotional tendency of comment data. If the emotional value was greater than 0, it was marked as positive, otherwise it was negative.

Table 3 is a statistical table of emotion analysis. It illustrates that before the plastic ban was fully enforced, the government promoted it through online platforms. At that time, in the netizens’ comment data, the average value of the overall emotion was 1.55, where the positive emotion average was 3.84, and the negative emotion average was 3.84. The average emotional value was −2.47. Furthermore, 63.62% of the netizens held a positive attitude, which shows that the netizens supported the new version of the “plastic ban”, while 36.24% of the netizens held a negative attitude, mainly thinking that it would have the same enforcement results as the “plastic ban” launched in 2008 which had not achieved long-term effective results. In addition, some netizens also believed that a “one-size-fits-all” approach should not be adopted until a suitable alternative product is developed. Besides, according to the content of Weibo topics, after the new version of the plastic restriction policy was fully enforced, netizens had extensive discussions on the alternatives to disposable plastic straws. According to the analysis results in Table 3, 71.11% of netizens held a negative attitude, mainly because they believed that disposable plastic straw substitutes have poor performance and tend to be soft, papery, and impossible to bite when drinking hot drinks. Through the analysis of online comment data, we can see that (1) the public supported the plastic restriction policy, which had a positive effect on promoting its full enforcement; (2) although netizens have a positive attitude towards the new version of the “plastic ban”, consumers feel badly about using paper drinking straws, which arouses their negative emotions.

## 6. Game-Based Model Construction and Simulation Analysis of the Multi-Stakeholder Strategy under the Plastic Straw Ban

Compared with the production of paper straws, companies need to invest more in the development and production of new plastic straw replacement products. For example, production of biodegradable straws (such as PLA straws) requires more manpower, materials, and financial resources. However, compared to the impact of paper straws on the environment, R&D of new products causes less pollution to the environment, and from the perspective of consumers, paper straws cannot satisfy the consumers’ sense of experience. However, in practice, companies put profit first and may adopt production strategies that are not conducive to environmental and social development to obtain greater benefits. For example, companies promote the development of biodegradable plastic straws but actually produce paper straws or disposable nondegradable plastic straws. Therefore, in order to protect the environment, improve the relationship between environmental protection and economic development, and avoid corporate violations, the government needs to promote the effect of the plastic straw ban and drive companies to develop new products. In the policy enforcement process, the government should also supervise the effect. Based on this, this section constructs the government–enterprise evolutionary game model and the government–enterprise–consumer evolutionary game model, respectively.

**Figure 2:**
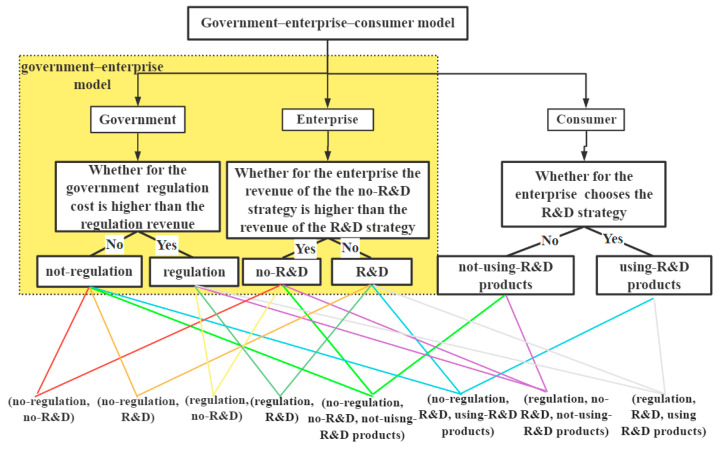
Structure of the government–enterprise game and the government–enterprise–consumer game.

At present, the Chinese government prohibits and restricts the use of disposable plastic straws for protecting the environment. Accordingly, enterprises have changed their production strategies in response to the call of the state. The production strategy of the enterprise is divided into the supply-level production strategy and the demand-level production strategy. The former is that a company chooses the production strategy based on its own production capacity. The latter is that it not only considers its own production capacity, but also takes the needs of consumers into account to further plan the production strategy. Therefore, the government–enterprise model constructed in this section starts from the supply level of the enterprise and aims to explore how the government measures the effect of the production strategy of the enterprise. Subsequently, on this basis, consumer acceptance and online comment factors are introduced, and from the demand level of the enterprise, a government–enterprise–consumer model is constructed to explore the changes in the production strategy of enterprises under the synergistic effect of the government and consumers, as well as the influence of consumer acceptance and online comments on their production strategies. Figure 2 shows the structure of the government–enterprise game and the government–enterprise–consumer game. The parameters involved in the models are shown in Table 4.

### 6.1. Government–Enterprise Evolutionary Game Model

#### 6.1.1. Problem Description and Assumptions

The government is an agent of the public. In order to promote social development, the government formulates and promotes all policies that are beneficial to the country and society. For example, in order to protect the environment, the government has enforced a ban on disposable nondegradable plastic straws. Manufacturing companies are the object of the policy, taking on social responsibilities such as reducing plastic production and researching and developing plastic substitutes in the production process. In addition, from an environmental perspective, disposable goods are harmful to the environment regardless of their materials [38], but due to their different materials and production methods, their environmental hazards are also different. Here, it is assumed that the new developed products are less harmful to the environment. However, companies seek profits and would not take the initiative to carry out technological innovation, i.e., will not adopt research and development strategies. The government is committed to achieving environmental protection goals and gives companies subsidies or tax reduction to encourage technological innovation. In addition, in reality, in order to obtain greater profits, companies will have fraudulent behavior, i.e., they will claim developing new products, but in fact they will adopt no-R&D strategies or produce substandard products. At this time, the government will impose relevant fines. Based on this, in the context of the enforcement of the ban on disposable nondegradable plastic straws, the measures that the government and enterprises may take during the game and the revenues and costs for both parties can be assumed as follows:

**Hypothesis** **1** **(H1).** *Based on the evolutionary game theory, both the government and enterprises are bounded rational subjects [11]. Assuming that the government strategy set is (regulation, no-regulation), the probability of the government choosing regulation is x, and the probability of choosing no-regulation is 1 − x (x ∊ [0,1]). The company strategy set is (R&D, no-R&D). If a company chooses the R&D strategy, it will produce and develop new products (such as PLA straws, PP straws); if a company chooses the no-R&D strategy, it will produce paper straws. The probability of the company choosing R&D is y, and the probability of the company choosing no-R&D is 1 − y (y ∊ [0,1]).*

**Hypothesis** **2** **(H2).** *By choosing regulation, the government will pay the corresponding cost C_g_ and obtain reputation revenue R_g_*_4_*. By choosing R&D, an enterprise will pay the corresponding cost C_e_*_1_*, obtain government subsidy S and revenue R_e_*_1_*, and the government will also obtain revenue R_g_*_1_* (e.g., environmental revenue). By choosing no-R&D, an enterprise will pay the corresponding cost C_e*2*_ to produce paper products, obtain revenue R_e*2*_, and the government will obtain revenue R_g_*_2_*. At the same time, assuming that R&D products are less harmful to the environment, enterprises choose different production strategies, production costs are different, and the government obtains different revenue, then C_e_*_1_* > C_e_*_2_*, R_g_*_1_* > R_g_*_2_**.

**Hypothesis** **3** **(H3).** *If an enterprise chooses the no-R&D strategy, it may have fraudulent behavior at this time, i.e., apply for R&D subsidies but actually choose the no-R&D strategy. Assuming that the probability of fraud is θ, the probability that the government will find fraud is β, and the amount of penalties collected by the government is P.*

#### 6.1.2. Basic Model

Based on the above assumptions, the government–enterprise revenue matrix is constructed as shown in Table 5.

The evolutionary game theory mainly describes the process of continuous optimization of the selection strategy of the subject with bounded rationality. Its basic idea is to study the degree of adaptation of the strategy and whether it is stable to resist the invasion of other strategies by analyzing the replication dynamic equations [9]. Based on the revenue matrix in Table 5, the average expected revenue of each entity can be calculated, and the dynamic equation for replication is as follows:

Assuming the expected revenue of choosing regulation is *E*_*g*1_, the expected revenue of choosing no-regulation is *E*_*g*2_, and the average expected revenue of the government is $\bar{E_{g}}$.

The government’s expected revenue of choosing regulation is:

(1) $$E_{g1}=y(R_{g1}+R_{g4}-S-C_{g})+(1-y)[R_{g2}+R_{g4}-C_{g}+\theta [\beta P-(1-\beta )S]$$

The government’s expected revenue of choosing no-regulation is:

(2) $$E_{g1}=y(R_{g1}-S)+(1-y)(R_{g2}-\theta S)$$

The average expected revenue of the government is:

(3) $${\bar{E}}_{g}=xE_{g1}+(1-x)E_{g2}$$

The government’s replication dynamic equation is:

(4) $$\begin{array}{ll} F(x) & =\frac{dx}{dt}=x(E_{g1}-{\bar{E}}_{g})=x(1-x)(E_{g1}-E_{g2})\\ & =x(1-x)[R_{g4}-C_{g}-\theta \beta (P+S)+y\theta \beta (P+S)] \end{array}$$

An enterprise’s replication dynamic equation is:

(5) $$F(y)=\frac{dy}{dt}=y(1-y)[(R_{e1}+S-C_{e1})-(R_{e2}+\theta S-C_{e2})+x\theta \beta (P+S)]$$

#### 6.1.3. Analysis of Evolution of a Stability Strategy

This section investigates the strategy of the government and enterprises based on their replicator dynamic Equations (4) and (5).

Assuming *F*(*x*) = 0, *F*(*y*) = 0, to obtain five local equilibrium points of the game system between the government and the enterprise (0,0), (0,1), (1,0), (1,1), (x*,y*) = $\left(\frac{\left(R_{e2}\;+\;S\;-\;C_{e2})-(R_{e1}\;+\;\theta S\;-\;C_{e1}\right)}{\theta \beta (P\;+\;S)},\frac{\theta \beta (P\;+\;S)\;-\;C_{g}\;-\;R_{g4}}{\theta \beta (P\;+\;S)}\right)$, where the equilibrium point (0,1) indicates the no-regulation and the R&D strategy. Based on realistic conditions, when the government chooses no-regulation, companies follow the market rules to compete freely, and the purpose of a company is to maximize the benefits, so it is inevitable to take the no-R&D strategy. Therefore, (0,1) cannot be a stability strategy and needs to be discarded. According to the stability analysis method of the Jacobian matrix for local equilibrium points proposed by Friedman [39], Jacobian matrix $J_{1}$ was established, and local stability analysis was performed, and the *DetJ*_1_ and *TrJ*_1_ values of four local equilibrium points were obtained. When *TrJ*_1_ < 0 and *DetJ*_1_ > 0, this point is stable, as shown in Table 6:

(6) $$J_{1}=\left[\begin{array}{cc} \frac{\partial F\left(x\right)}{\partial x} & \frac{\partial F\left(x\right)}{\partial y}\\ \frac{\partial F\left(y\right)}{\partial x} & \frac{\partial F\left(y\right)}{\partial y} \end{array}\right]$$

When *C_g_ − R_g_*_4_ > 0, (*R_e_*_2_ *− C_e_*_2_) − (*R_e_*_1_ *− C_e_*_1_) − (1 − *θ*)*S* > 0, i.e., the government regulation cost is higher than the regulation revenue, and the company does not research and develop products. At this time, the revenue of producing paper straws is higher than R&D costs. In this case, the no − regulation, the no-R&D strategy is stable.

When *C_g_ − R_g_*_4_ + *θβ*(*P* + *S*) < 0, (*R_e_*_2_ *− C_e_*_2_) − (*R_e_*_1_ *− C_e_*_1_) − *θβ*(*P* + *S*) − (1 − *θ*)*S* > 0, i.e., the revenue of no-R&D is lower than the revenue of R&D, the company has a fraudulent behavior, and the government regulation revenue is higher than the regulation cost. In this case, the regulation, the no − R&D strategy is stable.

When *C_g_ − R_g_*_4_ < 0, (*R_e_*_2_ *− C_e_*_2_) − (*R_e_*_1_ *− C_e_*_1_) − (1 − θ)*S* < 0, i.e., the regulation cost is lower than the regulation revenue, and the revenue of no-R&D is lower than the revenue of R&D. In this case, the regulation, the R&D strategy is stable.

#### 6.1.4. Simulation Analysis

**Figure 3:**
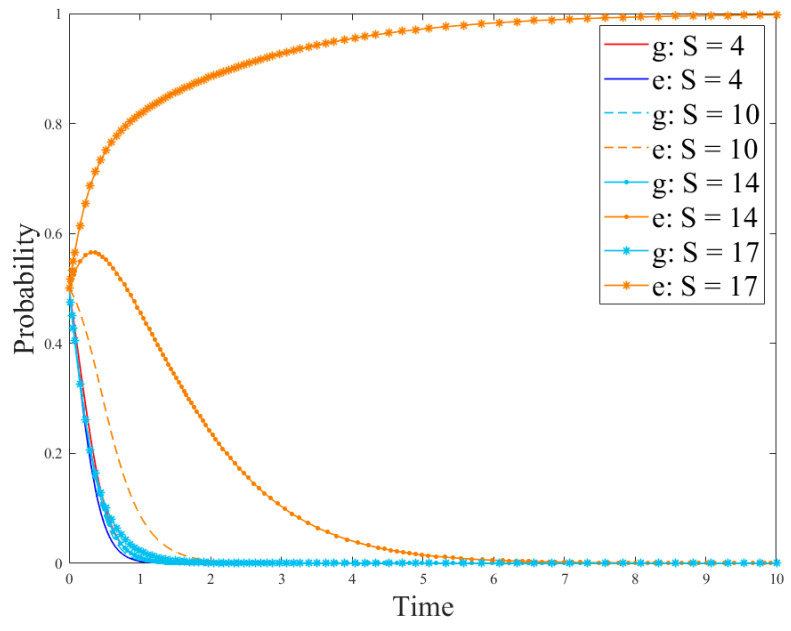
Evolution process of the government (*g*) and enterprises (*e*) with different government subsidy *S (S*:production subsidies or tax-free subsidies obtained by enterprises investing in research and development).

**Figure 4:**
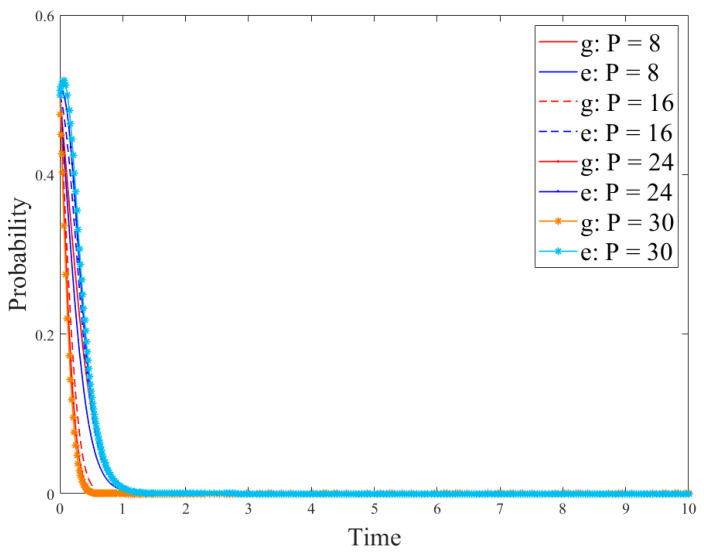
Evolution process of the government (*g*) and enterprises (*e*) with different government penalty *P (P*: penalty imposed by the government on enterprises that have cheated or produced substandard products).

Based on the conditions of the above stabilization strategy, the model parameters were set as follows: *R_g_*_4_ = 8; *C_g_* = 10; *P* = 8; *S* = 1; *R_e_*_1_ = 9; *R_e_*_2_ = 15; *C_e_*_1_ = 3; *C_e_*_2_ = 1; *θ* = 0.5; *β* = 0.5, the initial probability of the government and enterprises was set to *x = y* = 0.5. At this time, the cost of government regulation is lower than the revenue, and the revenue of no-R&D is higher than the revenue of R&D. The regulation, the no-R&D strategy is stable. Parameters *P* and *S* could be changed to discuss the government’s driving role in enterprise technological innovation. The results are shown in Figure 3 and Figure 4.

Figure 3 and Figure 4 show the evolution of the government and enterprise strategies after changing government subsidies and penalties, respectively. These two figures demonstrate that government subsidies can incentivize enterprises to carry out technological innovation and adopt R&D strategies. However, government penalties cannot effectively stop enterprises’ fraudulent behaviors or other irregularities. This is because if enterprises have higher revenue from producing paper straws than from developing new products, they will be more inclined to produce paper straws. In addition, if the government’s regulation costs are high, they will lead to few supervision effects, and only fines will be imposed on companies that violate the regulations. This will not effectively stop the violations of the companies and will damage the adoption of R&D strategies. At the same time, the interests of enterprises force them to choose no-R&D strategies.

### 6.2. Government–Enterprise–Consumer Evolutionary Game Model

#### 6.2.1. Problem Description and Assumptions

In the government–enterprise evolutionary game model, the government grants subsidies and benefits to enterprises, which encourage enterprises to innovate and invest funds in the research and development of new products, thereby effectively prohibiting the production of plastic straws and promoting alternatives. However, the data mined from Weibo comments shows that paper straws cannot satisfy the consumers’ sense of experience. Therefore, when companies develop new products, they should consider the consumers’ sense of experience and incorporate consumer acceptance of alternative products into production decisions. Based on this, this section constructs a government–enterprise–consumer evolutionary game model. Based on the assumptions of the government–enterprise model, the following assumptions are added:

**Hypothesis** **4** **(H4).** *Assuming that the consumer strategy set is (using-R&D products, not-using-R&D products), the probability of consumers using R&D products is z, and the probability of not usingR&D products is 1 − z (z ∊ [0,1]). The consumer’s utility of using new products is U*_1_ *and the utility of using paper products is U*_2_ *. When there is no product in the market that meets the consumers’ psychological expectations, they (consumers) have an acceptance for alternative products. Assuming that the consumer acceptance of R&D products is L, the acceptance of paper products is 1 − L (L ∊ [0,1]).*

**Hypothesis** **5** **(H5).** *According to Maslow’s hierarchy of needs theory, netizens will choose to express their opinions on Internet platforms in order to gain attention from others, express their social demands, gain respect from others, and gain emotional resonance. Assuming that no matter which product the consumer uses, the probability of commenting on the network platform is γ, the consumer’s own income is R_c_.*

**Hypothesis** **6** **(H6).** *Consumers post their views on the online platform, which promotes environmental protection publicity, so it brings benefits R_g_*_3_*to the government. Consumers’ participation in online reviews of enterprise products gives publicity to enterprises. Accordingly, enterprises obtain revenue R_e_*_3_*. However, consumers have different evaluations of different products, and the impact on enterprises is also different. Assuming that the impact of online reviews of new R&D products on enterprises is u*_1_*, the impact of online reviews of paper products on enterprises is u*_2_.

#### 6.2.2. Basic Model

Based on the above assumption, the government–enterprise–consumer revenue matrix was established, as shown in Table 7:

In the evolutionary game theory, replication dynamic equation describes the probability trend of a strategy over time. Based on the above revenue matrix, the average expected revenue of each entity can be calculated, and then the replication dynamic equation can be obtained.

The government’s expected revenue of choosing regulation is *E_g_*_1_, the expected revenue of choosing no-regulation is *E_g_*_2_, and the average expected revenue is $\bar{E_{g}}$.

The government’s expected revenue of choosing regulation is:

(7) $$\begin{array}{ll} E_{g1} & =yz(R_{g}{}_{1}+\gamma R_{g3}+R_{g}{}_{4}-S-C_{g})+y(1-z)(R_{g}{}_{1}+\gamma R_{g3}+R_{g}{}_{4}-S-C_{g})\\ & +z(1-y)[R_{g}{}_{2}+\gamma R_{g3}+R_{g}{}_{4}-C_{g}+\theta [\beta P-(1-\beta )S]]\\ & +(1-y)(1-z)[R_{g}{}_{2}+\gamma R_{g3}+R_{g}{}_{4}-C_{g}+\theta [\beta P-(1-\beta )S]] \end{array}$$

The government’s expected revenue of choosing no-regulation is:

(8) $$\begin{array}{ll} E_{g2} & =yz(R_{g}{}_{1}+\gamma R_{g3}-S)+z(1-y)(R_{g}{}_{2}+\gamma R_{g3}-\theta S)\\ & +y(1-z)(R_{g}{}_{1}+\gamma R_{g3}-S)+(1-z)(1-y)(R_{g}{}_{2}+\gamma R_{g3}-\theta S) \end{array}$$

The government’s average expected revenue is:

(9) $${\bar{E}}_{g}=xE_{g1}+(1-x)E_{g2}$$

The reproducible dynamic equation of the government is:

(10) $$\begin{array}{ll} F(x) & =\frac{dx}{dt}=x(E_{g1}-{\bar{E}}_{g})=x(1-x)(E_{g1}-E_{g2})\\ & =x(1-x)[-\theta \beta (P+S)y+R_{g4}-C_{g}+\theta \beta (P+S)] \end{array}$$

An enterprise’s expected revenue of choosing R&D is *E_e_*_1_, the expected revenue of choosing no-R&D is *E*_e2_, and the average expected revenue is $\bar{E_{e}}$.

At this moment, an enterprise’s expected revenue of choosing R&D is:

(11) $$\begin{array}{ll} E_{e1} & =xz[R_{e1}+\gamma (2u_{1}-1)R_{e3}+S-C_{e}{}_{1}]+(1-x)(1-z)L[R_{e1}+\gamma (2u_{1}-1)R_{e3}+S-C_{e}{}_{1}]\\ & +x(1-z)L[R_{e1}+\gamma (2u_{1}-1)R_{e3}+S-C_{e}{}_{1}]+z(1-x)[R_{e1}+\gamma (2u_{1}-1)R_{e3}+S-C_{e}{}_{1}] \end{array}$$

An enterprise’s expected revenue of choosing no-R&D is:

(12) $$\begin{array}{ll} E_{e2} & =xz(1-L)[R_{e2}+\gamma (2u_{2}-1)R_{e3}-C_{e}{}_{2}-\theta [\beta P-(1-\beta )S]]\\ & +x(1-z)[R_{e2}+\gamma (2u_{2}-1)R_{e3}-C_{e}{}_{2}-\theta [\beta P-(1-\beta )S]]\\ & +z(1-x)(1-L)[R_{e2}+\gamma (2u_{2}-1)R_{e3}-C_{e}{}_{2}+\theta S]\\ & +(1-x)(1-z)[R_{e2}+\gamma (2u_{2}-1)R_{e3}-C_{e}{}_{2}+\theta S] \end{array}$$

An enterprise’s average expected revenue is:

(13) $${\bar{E}}_{e}=yE_{e1}+(1-y)E_{e2}$$

Assuming *Q*_1_ = *R*_*e*1_ + *γ*(2*u*_1_ − 1)*R*_*e*3_ + *S − C*_*e*1_, *Q*_2_ = *R*_*e*2_ + *γ*(2*u*_2_ − 1)*R*_*e*3_ + *θS − C*_*e*2_, the reproducible dynamic equation of an enterprise is:

(14) $$\begin{array}{ll} F(y) & =\frac{dy}{dt}=y(E_{e1}-{\bar{E}}_{e})=y(1-y)(E_{e1}-E_{e2})\\ & =y(1-y)[Q_{1}(z+L-zL)-(Q_{2}-\theta \beta x(P+S))(1-zL)] \end{array}$$

The expected revenue of consumers using the new products is *E_c_*_1_, the expected revenue of not using the new products is *E_c_*_2_, and the average expected revenue of consumers is $\bar{E_{c}}.$

At this time, the expected revenue of consumers using the new products is:

(15) $$E_{c1}=xy(U_{1}+\gamma R_{c})+x(1-y)(1-L)\left(U_{2}+\gamma R_{c}\right)+(1-x)y(U_{1}+\gamma R_{c})+(1-x)(1-y)(1-L)\left(U_{2}+\gamma R_{c}\right)$$

The expected revenue of consumers not using the new products is:

(16) $$E_{c2}=xyL(U_{1}+\gamma R_{c})+x(1-y)\left(U_{2}+\gamma R_{c}\right)+Ly(1-x)(U_{1}+\gamma R_{c})+(1-x)(1-y)\left(U_{2}+\gamma R_{c}\right)$$

The average expected revenue of consumers is:

(17) $${\bar{E}}_{c}=zE_{c1}+(1-z)E_{c2}$$

The reproducible dynamic equation of consumers is:

(18) $$\begin{array}{ll} F(z) & =\frac{dz}{dt}=z(E_{c1}-{\bar{E}}_{c})=z(1-z)(E_{c1}-E_{c2})\\ & =z(1-z)[[U_{1}+\gamma R_{c}-(U_{1}-U_{2})L]y-(U_{2}+\gamma R_{c})L] \end{array}$$

#### 6.2.3. Analysis of Evolution of a Stability Strategy

Based on Equations (10), (14), and (18), the dynamic system equation of this game model is as follows:

(19) $$\left\{ \begin{array}{l} F(x)=x(1-x)[-\theta \beta (P+S)y+R_{g4}-C_{g}+\theta \beta (P+S)]\\ F(y)=y(1-y)[Q_{1}(z+L-zL)-(Q_{2}-\theta \beta x(P+S))(1-zL)]\\ F(z)=z(1-z)[[U_{1}+\gamma R_{c}-(U_{1}-U_{2})L]y-(U_{2}+\gamma R_{c})L] \end{array}\right.$$

Assuming F(x)=F(y)=F(z)=0, it can be seen that there are eight local equilibrium points in the three-dimensional dynamic system, i.e., (0,0,0), (1,0,0), (0,1,0), (0,0,1), (1,1,0), (1,0,1), (0,1,1), (1,1,1). However, these eight strategy combinations are not necessarily all stability strategies. The equilibrium points (0,1,0) and (0,1,1) indicate (no-regulation, R&D, not-using-R&D products) and (no-regulation, R&D, using-R&D products), respectively. When the government chooses the no-regulation strategy, enterprises compete freely according to the market law. In order to obtain greater revenue, it is inevitable not to develop the R&D strategy, so (0,1,0) and (0,1,1) should be omitted. According to the Lyapunov system stability criterion, when the eigenvalues of the Jacobian matrix are negative, the corresponding equilibrium point is stable [40]. The Jacobian matrix *J*_2_ can be obtained from Equation (19) and the eigenvalues of the Jacobian matrix corresponding to each equilibrium point are shown in Table 8.

(20) $$J_{2}=\left[\begin{array}{ccc} \frac{\partial F\left(x\right)}{\partial x} & \frac{\partial F\left(x\right)}{\partial y} & \frac{\partial F\left(x\right)}{\partial z}\\ \frac{\partial F\left(y\right)}{\partial x} & \frac{\partial F\left(y\right)}{\partial y} & \frac{\partial F\left(y\right)}{\partial z}\\ \frac{\partial F\left(z\right)}{\partial x} & \frac{\partial F\left(z\right)}{\partial y} & \frac{\partial F\left(z\right)}{\partial z} \end{array}\right]$$

When the stability conditions of the above strategies are met, by analyzing the eigenvalues of the Jacobian matrix at the equilibrium point, it can be seen that the three-dimensional dynamic system has three equilibrium points: (0,0,0), (1,0,0), (1,1,1). The following analysis is made on the conditions that meet the three stability strategies:

**Scenario** **1.** *When R_g_*_4_ *+ θβ(P + S) < C_g_ and LQ*_1_ *< Q*_2_ *, the government regulation cost is greater than the revenue obtained by the government after taking the regulation measures, and the revenue obtained by an enterprise choosing the no-R&D strategy is greater than the revenue obtained by an enterprise choosing the R&D strategy. At this time, (no-regulation, no-R&D, not-using-R&D products) is the stability strategy after the game.*

**Scenario** **2.** *When R_g_*_4_ *+ θβ(P + S) > C_g_ and LQ*_1_ *+ θβ(P + S) < Q*_2_ *, the government regulation cost is less than the revenue obtained after the government takes the regulation measures, and the revenue obtained by an enterprise choosing the no-R&D strategy is greater than that obtained by an enterprise choosing the R&D strategy. At this time, (regulation, no-R&D, not-using-R&D products) is the stability strategy after the game.*

**Scenario** **3.** *When R_g_*_4_ *> C_g_ and Q*_2_ *− θβ(1 − L)(P + S) < Q*_1_ *, the government regulation cost is less than the revenue obtained after the government takes the regulation measures. At the same time, the revenue obtained by an enterprise choosing the R&D strategy is greater than that obtained by an enterprise choosing the no-R&D strategy. At this time, (regulation, R&D, using-R&D products) is the stability strategy after the game.*

#### 6.2.4. Simulation Analysis

**Figure 5:**
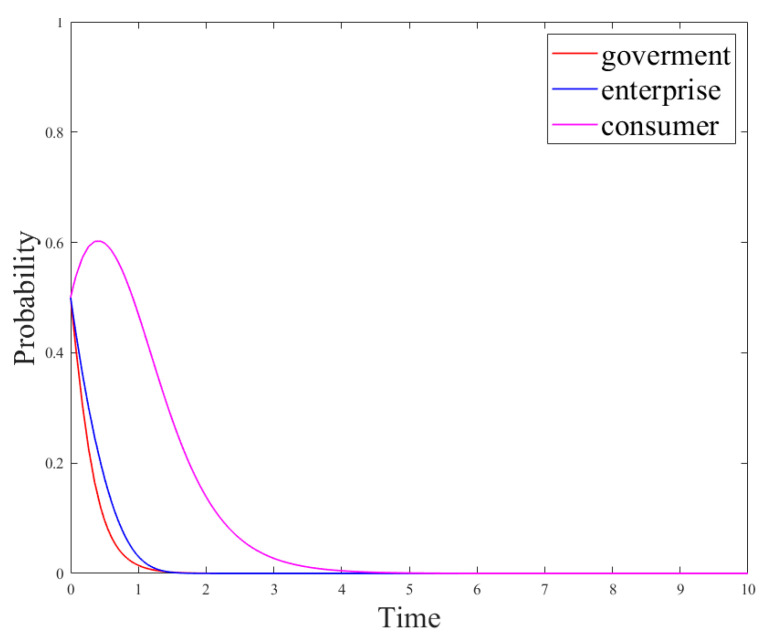
Evolution results under Scenario 1.

This section uses the MATLAB software to simulate the aforementioned evolutionary game model to explore the evolutionary game process of each subject’s strategy under the factors of consumer acceptance and network comments on plastic straw substitutes. Firstly, we analyzed the evolution results of the subject’s strategies in different situations. Secondly, we discussed the impact of the subject’s initial strategy, government measures, network comments, and consumer acceptance on the results of the evolutionary game. Notice that in this section, for the three different scenarios in the analysis of the government–enterprise–consumer stability strategy, the parameters were set as follows:

**Scenario** **1.** *When the government regulation cost and the enterprise R&D cost are high, set the parameters of the evolutionary game model as follows: R_g_*_4_*= 4, C_g_ = 10, P = 8, S = 1, R_e_*_1_*= 9, R_e_*_2_*= 15, R_e_*_3_*= 8, R_c_ = 5, C_e_*_1_  *= 3, C_e_*_2_ *= 1, U*_1_ *= 5, U*_2_ *= 1, u*_1_ *= 0.5, u*_2_ *= 0.28, L = 0.5, θ = 0.5, β = 0.5, γ = 0.5. The system stability strategy is (no-regulation, no-R&D, not-using-R&D products), and its evolution path is shown in Figure 5.*

**Figure 6:**
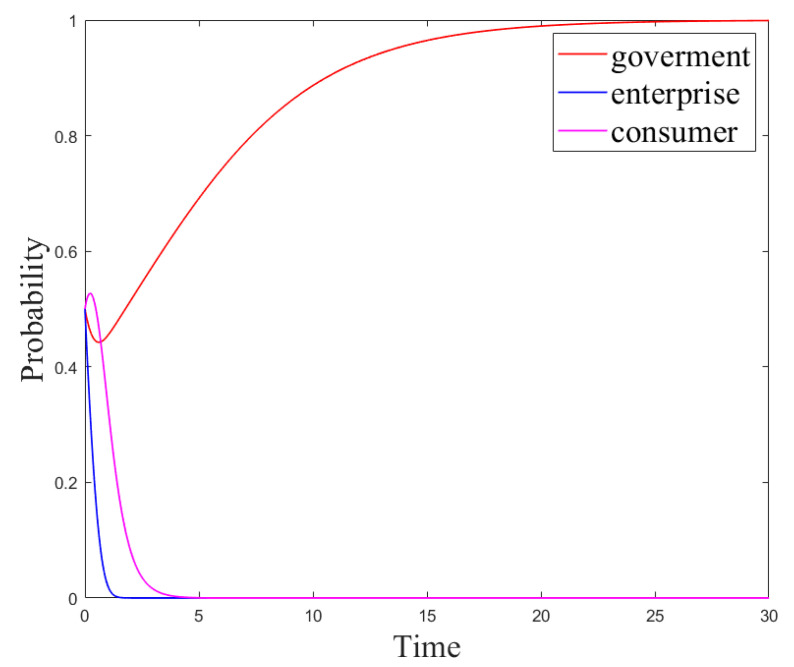
Evolution results under Scenario 2.

**Scenario** **2.** *When the government regulation revenue and the enterprise R&D cost are high, set the parameters of the evolutionary game model as follows: R_g_*_4_ *= 8, C_g_ = 10, P = 8, S = 1, R_e_*_1_ *= 9, R_e_*_2_ *= 15, R_e_*_3_ *= 8, R_c_ = 5, C_e_*_1_ *= 3, C_e_*_2_ *= 1, U*_1_ *= 5, U*_2_ *= 1, u*_1_ *= 0.5, u*_2_ *= 0.28, L = 0.5, θ = 0.5, β = 0.5, γ = 0.5. The system stability strategy is (regulation, no-R&D, not-using-R&D products), and its evolution path is shown in Figure 6.*

**Figure 7:**
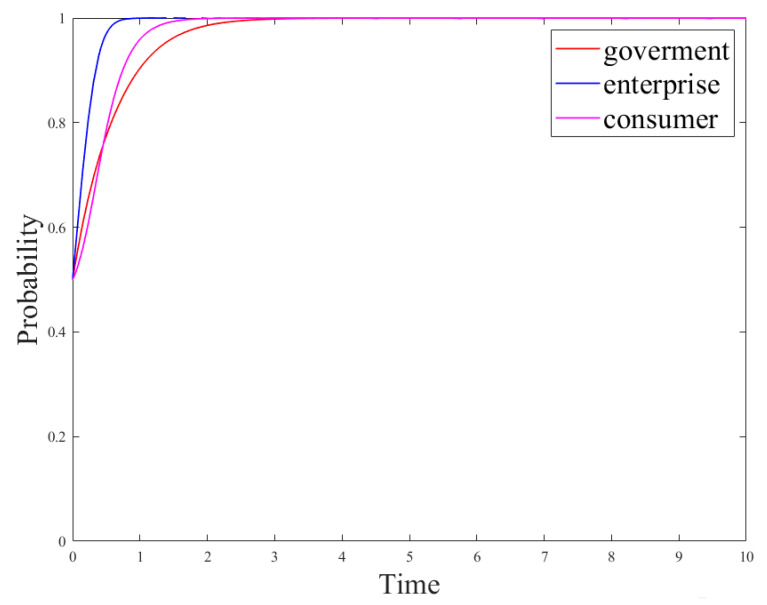
Evolution results under Scenario 3.

**Scenario** **3.** *When the government regulation revenue and the enterprise R&D revenue are high, set the parameters of the evolutionary game model as follows: R_g_*_4_*= 12, C_g_ = 10, P = 8, S = 1, R_e_*_1_*= 15, R_e_*_2_*= 9, R_e_*_3_*= 8, R_c_ = 5, C_e_*_1_*= 3, C_e_*_2_ *= 1, U*_1_*= 5, U*_2_*= 1, u*_1_*= 0.5, u*_2_*= 0.28, L = 0.5, θ = 0.5, β = 0.5, γ = 0.5. The system stability strategy is (no-regulation, no-R&D, not-using-R&D products), and its evolution path is shown in Figure 7.*

The system stability strategy under Scenario 3 is the most ideal strategy, yet Scenarios 1 and 2 aim to explore how the system strategy reaches the most ideal state. Therefore, in order to better explain the impact of the main parameters on the system strategy and how the main strategies achieve their ideal stability strategy, this section discusses the impact of the initial strategy on the system evolution results under Scenario 3, the impact of government measures on the system evolution results under Scenario 2, and the impact of network comments and consumer acceptance on the system evolution results under scenario 1.

(1) Analysis of the results of the subject’s strategy evolution in different situations

Figure 5, Figure 6 and Figure 7 are the evolution results under Scenario 1, Scenario 2, and Scenario 3, respectively. The final stability strategy combinations of the three scenarios are (0,0,0), (1,0,0), and (1,1,1). At this time, the evolution trend of the consumer strategy is consistent with that of the enterprise strategy, and the consumer strategy reaches a stable state more slowly than the enterprise strategy. The stability strategies of the government and enterprises mainly depend on their respective costs and revenues, but the consumer strategy mainly depends on the production strategy of the enterprise. Under the parameter settings of the three scenarios, the consumers’ revenue from using new R&D products is higher than that from using paper products. However, in the simulation results of Scenarios1 and2, the consumer’s stability strategy is not to use the new product, and only in Scenario 3, the consumer’s stability strategy is to use the new product. This is because straw products are a necessity for consumers, and which straw substitutes they choose is mainly determined by the enterprise’s production strategy. In addition, the speed wherewith the consumer strategy reaches a stable state is slower than that wherewith the enterprise reaches the stable state.

(2) Influence of the initial strategy on the evolution results

**Figure 8:**
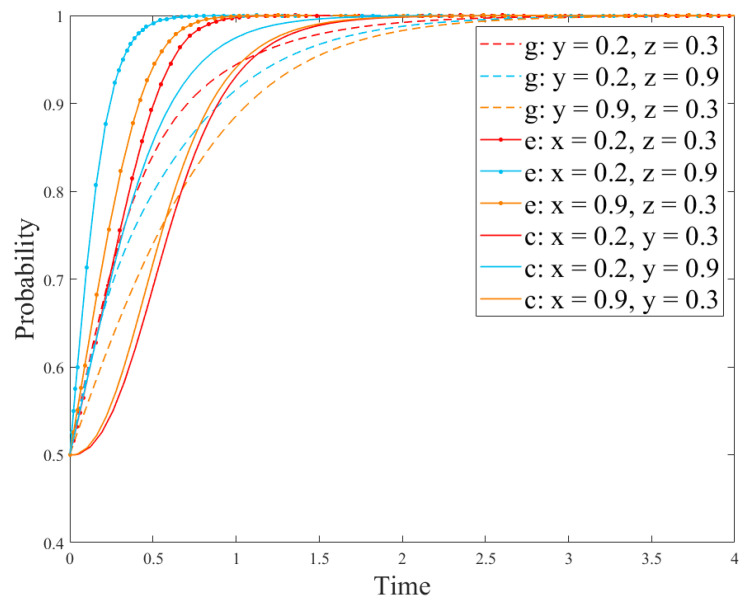
Evolution results of the system strategy with different subject’s initial strategy (g: government, e: enterprise, c: consumer).

Figure 8 shows the evolution results of a tripartite game where the probability values (*x*,*y*,*z*) of the initial strategies of the government, enterprises, and consumers were changed in Scenario 3. As can be seen in Figure 8, the greater the probability (*y*) of technological innovation and R&D of new products is, the slower the government’s regulation strategy reaches the stable state. This is because the greater probability of enterprises choosing the R&D strategy represents the smaller probability of fraud or production of unqualified products and the smaller effect of government regulation. However, compared with the government’s impact on an enterprise’s production strategy, consumers have a greater impact on it, i.e., the greater the value *z* is, the faster the enterprise chooses to develop new products to reach the stable state. This is because an enterprise is a profit-making organization which takes profit as the primary purpose and will quickly adjust its production strategy according to consumer demand. Meanwhile, according to the analysis results in Figure 5, Figure 6 and Figure 7, the consumer strategy is affected by the enterprise strategy. Furthermore, according to Figure 8, the higher the probability (*y*) of enterprises choosing R&D strategies, the faster the consumers choose to use the strategy of new products to reach the stable state.

(3) Impact of government’s measures on the evolution

**Figure 9:**
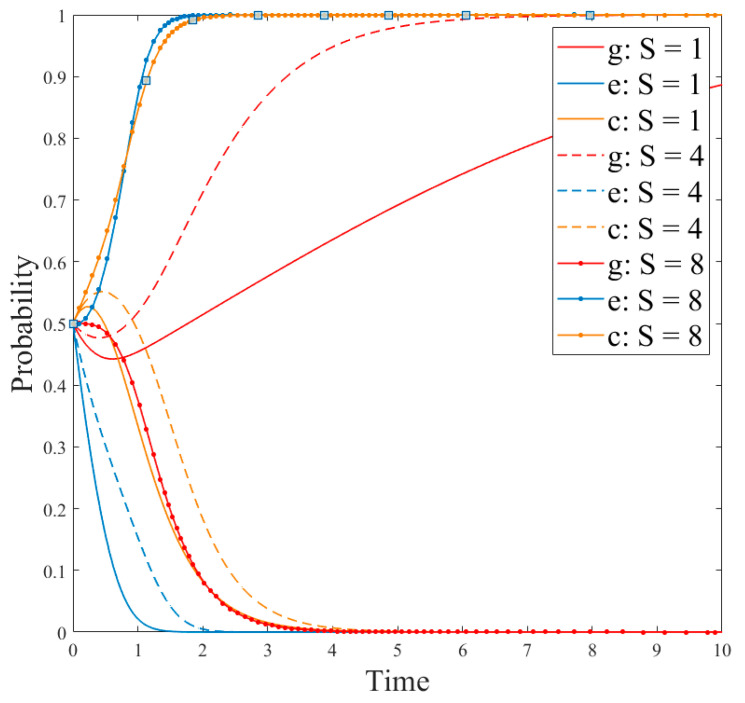
Evolution results of the system strategy with different subsidy(S) (*g*: government, *e*: enterprise, *c*: consumer) (*S*:production subsidies or tax-free subsidies obtained by enterprises investing in research and development).

**Figure 10:**
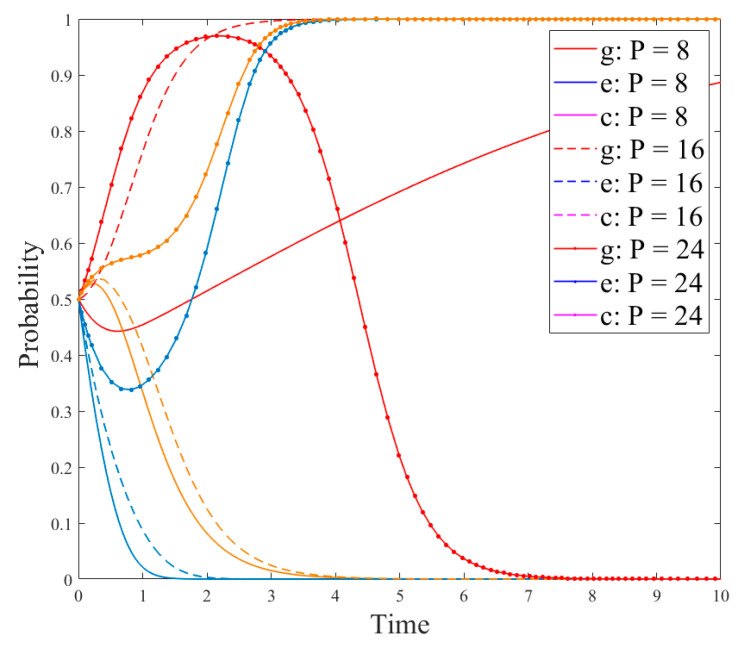
Evolution results of system strategy with different penalty(P) (*g*: government, *e*: enterprise, *c*: consumer) (*P*: penalty imposed by the government on enterprises that have cheated or produced substandard products).

Figure 9 and Figure 10 are the simulation results under Scenario 2. At this time, the system stability strategy is (regulation, no-R&D, not-using-R&D products), and its evolution path is shown in Figure 6. It can be seen in Figure 9 that under the stimulation of government subsidies, enterprises will change from the no-R&D strategy to the R&D one. Accordingly, the consumer strategy will change from notusing R&D products to using them. However, the government’s strategy has changed from regulation to no-regulation. The reason is that driven by government subsidies, production strategies of more enterprises have changed. Although this government measure can reduce the pressure on environmental pollution caused by plastic straw substitutes, the government regulation cost will also increase, resulting in a change of the government strategy. In addition, Figure 10 demonstrates that when the government increases the penalties for enterprises with fraudulent compensation behavior or producing unqualified products, it will urge enterprises to choose R&D strategies, which is different from the evolution results of Figure 4. The reason is that in the evolution process of Figure 10, enterprises consider the factor of consumer demands, and their production strategies are mainly affected by consumers. However, with the increase in the regulation cost, the government strategy will change from regulation to the no-regulation strategy. This is because with the increase of government regulation, although the cheating and compensation behavior of enterprises will be reduced, the government regulation cost will increase accordingly, resulting in the move from the regulation strategy to the no-regulation strategy.

(4) Impact of comments on the evolution

**Figure 11:**
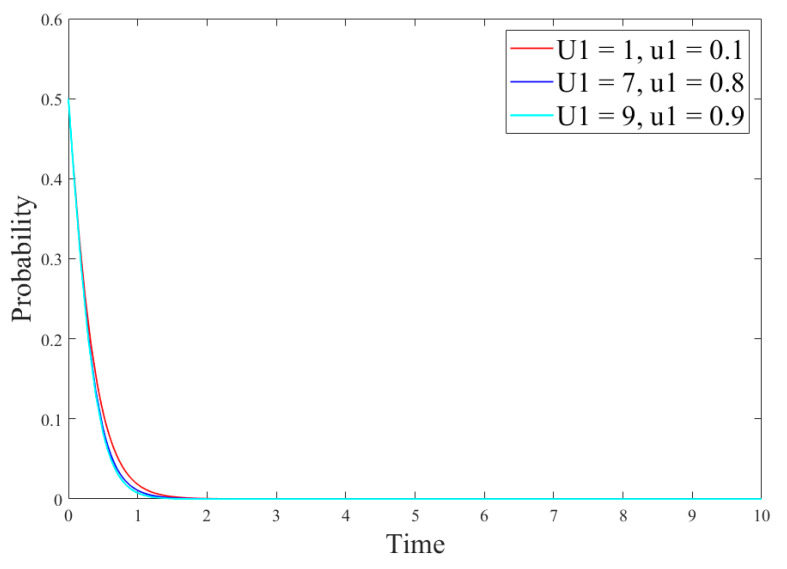
Evolution results of the government’s strategy.

**Figure 12:**
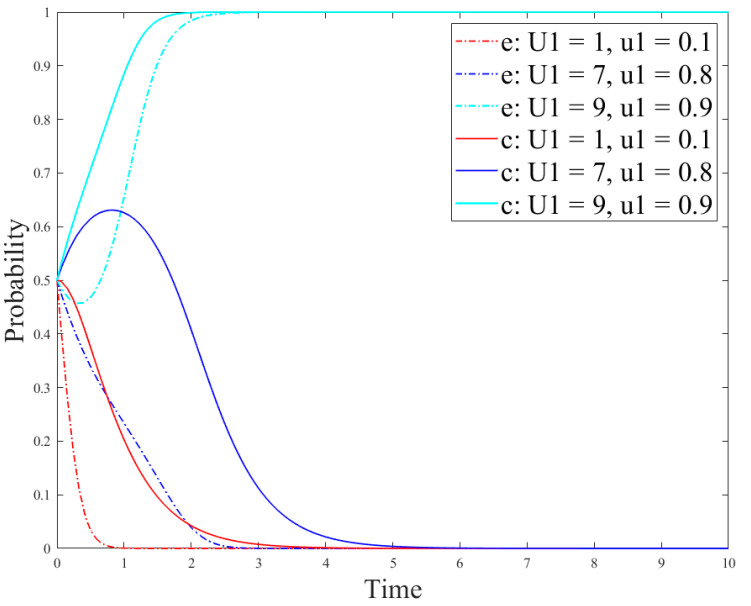
Evolutionresults of the enterprise and consumer strategies (*e*: enterprise, *c*: consumer) (*U*_1_:consumer’s utility of using new products; *u*_1_:degree of positive influence of the online public opinion on the new product on the company.).

Section 4 provides an emotion analysis of the online comments on paper straws. It was found that only 28.88% of the Internet users have a positive attitude towards paper straws replacing the traditional plastic straws. In view of the consumers’ negative attitude towards paper straws, this paper analyzes the impact of network comments on R&D of new products on the game system so as to explore the role of public network opinion on the R&D strategy. Since the utility obtained by consumers using new products is directly proportional to the positive attitude in new product online reviews, based on the parameter-setting conditions of scenario 1, our study adjusted the utility *U*_1_ obtained by consumers using new developedproducts and discussed the positive impact *u*_1_ of online reviews for newdeveloped products on enterprises at the same time so as to analyze the changes of strategies of different subjects. The results are shown in Figure 11 and Figure 12.

Figure 11 shows the impact of online comments on government’s strategies, and Figure 12 shows the impact of online comments on the evolution of the enterprise and consumer strategies. Figure 11 demonstrates that online comments have little impact on government’s strategies because the government is less disturbed by external factors and its strategy choice mainly depends on its cost–revenue ratio. In addition, as can be seen in Figure 12, the critical value *α* of *u*_1_ is between 0.8 and 0.9. When *u*_1_ < *α*, the larger value of *u*_1_ indicates the time for reaching the stable state in case of the no-R&D strategy and notusing R&D products is slower. When *u*_1_ > *α,* enterprises and consumers will change their strategies, in which the enterprise production strategy will change from the no-R&D strategy to the R&D one, and its R&D strategy will gradually reach the stable state. At this time, the consumer strategy will reach the stable state faster than the enterprise strategy. This is because the larger proportion of consumers who hold a positive attitude towards the new products indicates the higher acceptance of the new products, and their demands will be greater. At this time, an enterprise will adjust the production strategy according to the market demand and invest more to develop new products. Besides, in the following section, we explore the impact of consumer acceptance on the evolution results.

(5) Impact of consumer acceptanceon the evolution

**Figure 13:**
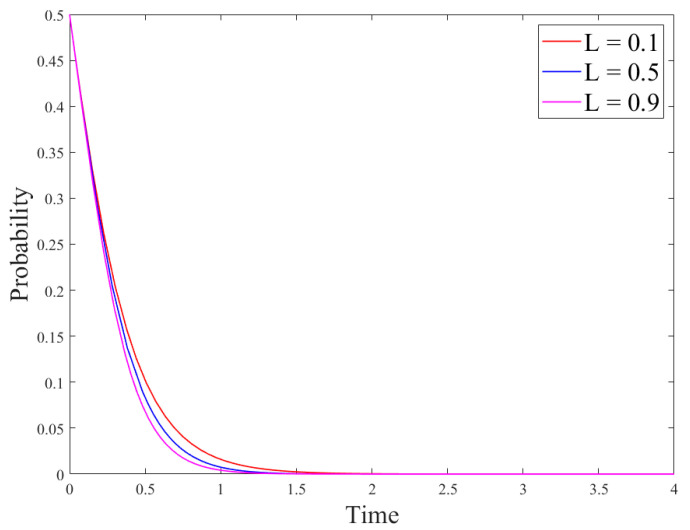
Evolution results of the government strategy. (*L*: consumer acceptance of new products).

**Figure 14:**
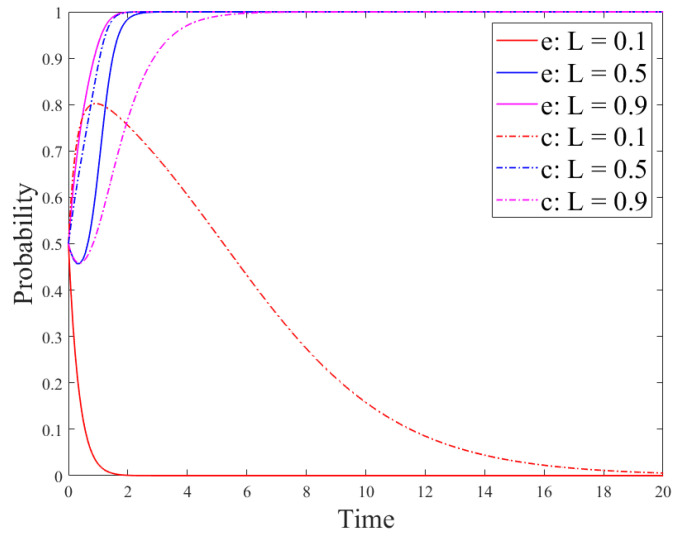
Evolution results of the enterprise and consumer strategies (*e*: enterprise, *c*: consumer).

In the simulation results in Figure 12, it is easy to find that when *u*_1_ is greater than its critical value *α*, the consumer strategy reaches the stable state faster than the enterprise strategy. However, according to the analysis results in Figure 7, the result is just the opposite. In order to analyze the reason, the parameter setting here is based on Section 4 and the consumer acceptance *L* is used to discuss the influence on the system evolution results. The analysis results are shown in Figure 13 and Figure 14.

Figure 13 and Figure 14 show the evolution results of the government, enterprise, and consumer strategies under different consumer acceptance. Figure 13 demonstrates that the greater value of *L* means the government strategy reaches the stable state faster. However, the government strategy has not evolved from the no-regulation strategy to regulation. The reason is that the government strategy is mainly affected by its own costs and benefits. Moreover, as can be seen in Figure 14, the critical value *l*_1_ of consumer acceptance is between 0.1 and 0.5. When *L* is less than *l*_1_, the stability strategies of enterprises and consumers are the no-R&D strategy and the not-using-R&D products strategy, respectively. On the contrary, when *L* is greater than *l*_1_, the stability strategies of enterprises and consumers are the R&D strategy and the using-R&D products strategy, respectively. In addition, when consumer acceptance is between 0.5 and 0.9, there also exists a critical value *l*_2_. When 0.5 < *L* < *l*_2_, the consumer strategy reaches the stable state faster than the enterprise strategy. When *L* > *l*_2_, the enterprise strategy reaches the stable state faster than the consumer strategy. This is because enterprises initially choose the no-R&D strategy; however, if consumers have greater acceptance for new products, they also have greater demands for them. Then, if an enterprise chooses the R&D strategy, consumers will tend to choose to use R&D products. Therefore, if other enterprises choose the R&D strategy, the number of enterprises choosing the R&D strategy will also increase, so that the enterprise the R&D strategy will reach the stable state faster than the consumer strategy.

## 7. Results and Discussions

In this section, we discussed three aspects, analysis of the simulation results, broader applications, and the limitations of this paper. The detailed content is illustrated as follows.

### 7.1. Analysis of the Simulation Results

Based on the simulation analysis, the following results could be obtained:

(1) Since straws are a necessity for consumers, the consumer strategy changes with the change of the enterprise’s production strategy. Based on this, enterprises play an indispensable role in the treatment of disposable plastic straws, and the government should do a good job of regulation.
(2) After enterprises choose the R&D strategy, the government will move from regulation to nonregulation under the pressure of regulation cost. However, in order to avoid the violation phenomenon due to the reduction of government regulation, after the enterprise the R&D strategy reaches stability, the government should continue to adopt regulation. Therefore, in order to promote effective enforcement of the ban on disposable plastic straws and achieve the purpose of protecting the ecological environment, the government should establish a long-term and effective regulation mechanism, regulate the whole production process of enterprises, and formulate financial support policies to encourage production enterprises to develop new products. At the same time, enterprises with violations should be severely punished.
(3) When *u*_1_ > α (0.8 < α < 0.9) and when *L* < *l*_1_(0.1 < *l*_1_ < 0.5) or *L* > *l*_2_(0.5 < *l*_2_ < 0.9), the enterprise strategy will reach the stable state faster than the consumer strategy. As a result, when formulating production strategies, enterprises should comprehensively consider environmental protection and consumer acceptance, pay attention to public network opinion on the products, understand the consumers’ demand for products, and provide references for product functionality and environmental protection. Furthermore, enterprises can guide the public network opinion to publicize new products and improve product popularity and consumers’ awareness of environmental protection. At this moment, the government should also take corresponding measures to improve the public’s awareness of environmental protection and stimulate enterprises to change their production strategies and reduce the production of plastic products from the perspective of consumers.

Considering the production and use of single-use plastic straws, this paper introduced the online comment factor, and then conducted a quantitative analysis of the game player strategies through numerical simulation experiments. Compared with the existing research on disposable plastic straw substitutes, scholars mainly studied functionality [2,21] from the perspective of consumers and carbon footprint [5,6] of plastic straw substitutes. Their results also focus on which disposable plastic straw substitutes are more functional and less polluting. However, in this paper, we mainly paid attention to which measures the government takes can encourage enterprises to develop alternatives of plastic straws with less environmental pollution and how enterprise strategies are affected by consumer strategies, and then which production strategies are adopted. In addition, we also find that the government will have an impact on the enterprise strategy, which is consistent with the conclusions of other studies [29,30]. Furthermore, the conclusion that enterprise strategies are affected by consumers is also consistent with the actual situation.

Furthermore, compared with the existing literature, this paper has the following innovations: (1) the influence of consumer acceptance of plastic substitutes and online comments on the profits of enterprises are considered; (2) from the supply side and the demand side, production strategies of enterprises are studied, which is helpful for comprehensively exploring the effective means to promote enterprise transformation; (3) it is not effective to control the growth of such enterprises only by imposing fines on them.

### 7.2. Broader Applications

Regulation of plastic pollution has gradually shifted from early bans of plastic bags and regulation of general waste handling toward facilitating the transition aimed at a circular economy. This shift in focus has resulted in legislation, and countries around the world have begun to focus explicitly on how much plastic waste they produce, how much is managed, and whether that management is sustainable, such as the European single-use plastics directive which aims at regulating plastics at several different stages in the lifecycle rather than focusing on one stage in the lifecycle, such as the waste phase [41].On 4 December 2019, Prime Minister of the Vietnamese Government issued the National Action Plan for the Management of Marine Plastic Litter by 2030. One of the general objectives in dealing with marine plastic litter in the Plan is to take an approach which aligns with the circular economy model, facilitating plastic waste collection, recycling, and reuse in Vietnam. The Plan also encourages organizations and individuals to increase recycling and reuse of plastic waste and promotes the development of a circular economy and green growth [42]. In July 2020, four departments, including the Ministry of Agriculture and Rural Affairs of China, promulgated the more targeted “Management Measures of Agricultural Mulch Film”. This measure regulates the production, sales, use, recycling, reuse, and supervision of agricultural mulch film. In the recycling process, the new measure requires farmers to recycle field mulch film waste before the expiration of the utilization period and hand it over to recycling outlets or other recycling works [43].Recycling or reuse of plastics in circulation is essential to prevent increased accidental or purposeful release of polymeric materials into the environment and thus curb environmental pollution. Nevertheless, these socio-material challenges necessitate a systematic approach to plastic waste management. It is imperative to maintain polymers in their highest value state, ensuring that the materials we depend upon stay in circulation. Thus, contamination of plastics, sorting, and degradation remain the major barriers to efficient recycling [44].

With regard to the abovementioned existing plastic circulation economy, scholars rarely consider how to encourage companies to develop recycling devices and how to encourage consumers to participate in the recovery process. Based on the findings of this paper, there are discussions as follows: during the plastic recovery process, enterprises have the responsibility of recovering the R&D plastic garbage, and consumers have an important role in participating in plastic recycling. In the process of promoting plastic recycling, the government should act as a supervisor while incentivizing companies and consumers to participate in the process of plastic recycling. It is difficult to recycle different types of plastic. However, in areas where plastic use is concentrated, recycling stations can be set up, e.g., in express delivery stations (express delivery is a disaster area of plastic pollution), and consumers can be encouraged to take outer packages from express delivery to recycling stations by giving them red envelopes, points, and other welfare measures. Recycled express packaging is still used in the production of express packaging. For example, garbage bags used in daily life are extremely difficult to recycle, but they are also a significant cause of plastic pollution. Manual recovery may not be feasible and sustainable, so the development of automatic separation devices can be considered to achieve mechanical recovery.

### 7.3. Limitations

This paper still has the following deficiencies and aspects to be studied further:

(1) This paper only studies the enterprise straw production strategy in the background of the plastic straw ban. In the follow-up study, our research should be extended to the enterprise production strategy for other plastic products in the context of the environmental regulation policy [45].
(2) When considering the impact of the consumer strategy on the enterprise strategy, consumers’ environmental awareness is not taken into account [46]. In fact, it affects their consumption behavior. As a result, it should be further explored in the future.

## 8. Conclusions

In the background of enforcing the new “plastic ban”, from the perspective of enterprise production, this paper constructed a government–enterprise game model from the supply level of enterprises to explore the impact of government measures on the strategies of production enterprises. Then, from the perspective of the enterprise demand level, based on the government–enterprise game model, this paper introduced consumer acceptance, constructed a government–enterprise–consumer game model, and discussed the changes of the enterprise production strategy with the participation of the government and consumers. Finally, through the simulation technology, this paper analyzed the evolution process of different subject strategies in different situations and discussed the impact of the subject’s initial strategy probability on the system strategy, as well as the influence of network comments and consumer acceptance on the evolution process of different subject strategies.

Based on the above analysis results, the following conclusions were made:

(1) The government strategy is less affected by external interference factors, and its change mainly depends on the government’s own cost–revenue ratio. The enterprise strategy and the consumer strategy influence each other. Compared with the influence of the government, the enterprise production strategy is more affected by consumers.
(2) The government has formulated measures to encourage enterprises to carry out technological innovation and develop alternatives to plastic straws. In order to ensure profitability, enterprises will quickly adjust their strategies when the external environment changes. At the same time, government subsidies stimulate enterprises to choose R&D strategies, but for enterprises with fraud, penalties to punish them will not be sufficient to achieve the desired results.
(3) The degree of positive influence of the online public opinion on new products on the enterprises and consumer acceptance have a decisive impact on whether enterprises choose R&D strategies.

## Figures and Tables

**Table 1:** Policies issued by the national, provincial, and municipal governments in China.

Level	Document	Content
National	Opinions on Further Strengthening Plastic Pollution Control (2020, No. 80)	Phased objectives and tasks formulated to prohibit and restrict the production, sales, and use of some plastic products. It is clear that the use of nondegradable disposable plastic straws would be prohibited in the catering industry nationwide by the end of 2020. By 2022, plastic alternative products will be promoted. By 2025, plastic pollution will be effectively controlled.
	Notice on Solidly Promoting Plastic Pollution Control	Detailed standards for the prohibition and restriction management of relevant plastic products published, covering plastic shopping bags, agricultural plastic mulch, disposable plastic tableware, disposable plastic straws, etc. The notice emphasizes strengthening the supervision and management of the production and sales of plastic products, catering, and other fields.
Beijing	Beijing Plastic Pollution Control Action Plan (2020–2025)	Plastic reduction targets and tasks formulated for six key industries such as catering, takeout platforms, and wholesale and retail. By the end of 2020, the city’s catering industry would prohibit the use of nondegradable disposable plastic straws. Strengthening of the supervision and management of plastic production enterprises and punishment of enterprises that produce plastics in violation of theregulations according to the law.
Anhui Province	Implementation Plan for Further Strengthening the Plastic Pollution Control in Anhui Province	It was required that by the end of 2020, the catering industry in the Anhui province prohibit the use of nondegradable disposable plastic straws. A detailed scheme for the promotion and application of plastic alternative products put forward.
Guangdong Province	Implementation Opinions on Strengthening the Plastic Pollution Control	For 2020, 2022, and 2025, the goals and tasks of the “plastic ban” were formulated, respectively. It was required that the catering industry in the province prohibit the use of nondegradable disposable plastic straws by the end of 2020 and put forward more detailed requirements for the application of alternative products.
Shanghai	Implementation Plan of Shanghai Municipality on Further Strengthening the Plastic Pollution Control	Focusing on the goal of prohibiting and restricting the production, sales, and use of some plastic products, this paper gives a specific scheme for the promotion and application of alternative products and modes and also makes corresponding requirements for the recycling and disposal of plastic waste.
Zhejiang Province	Implementation Measures on Further Strengthening the Plastic Pollution Control	It was required that by the end of 2020, shopping malls, supermarkets, pharmacies, bookstores, and other places in the built-up areas of Hangzhou, Ningbo, and Shaoxing be prohibited from using nondegradable plastic bags, and nondegradable disposable plastic straws be prohibited in the catering industry throughout the province.
	Implementation Plan of Hangzhou on Further Strengthening the Plastic Pollution Control	Prohibition of the production and sale of some plastic products. Special treatment actions for nondegradable plastic bags and disposable plastic products. It was required that by the end of 2020, the use of nondegradable disposable plastic straws be prohibited in the catering industry in the whole city. A detailed scheme for the recycling and disposal of plastic products was provided.

**Table 2:** Weibo topics and comments after filtering.

Type	Topic	Comments	Proportion
Before the policy enforcement (19 January 2020–30 December 2020)	Plastic straws will be banned by the end of the year	11,744	82.08%
	Some plastic products will be banned or restricted	284	1.98%
	Nondegradable disposable plastic straws are prohibited in the catering industry	381	2.66%
	Nondegradable disposable plastic straws prohibited in Shanghai	886	6.19%
	Shanghai’s new plastic limit	680	4.75%
	Chengdu’s strongest plastic limit order is coming	332	2.32%
After the policy enforcement (1 January 2021–30 April 2021)	Consumers complain about paper straws for milk tea	19,585	47.6%
	KFC uses wooden spoons	11,295	27.45%
	First day of the plastic straw ban	1524	3.7%
	Happiness deprived by paper straws	411	0.99%
	Why I hate paper straws	8326	2.02%

**Table 3:** Emotional analysis.

Statistical Indicators	Before the Enforcement	After the Enforcement
Proportion of positive emotional comments	63.62%	28.88%
Proportion of negative emotional comments	36.24%	71.11%
Average positive emotion	3.84	2.63
Average negative emotion	−2.47	−2.37
Average overall emotion	1.55	−0.92

**Table 4:** Parameters involved in the model (g: government; e:enterprise; c: consumer).

Parameters	Description
*R_g_*_1_	Government’s revenue from the production and research and development of new products by enterprises (*R*_*g*1_ > *R*_*g*2_)
*R_g_*_2_	Government’s revenue from the production of paper products by enterprises
*R_g_*_3_	Government’s revenue from consumers’ participation in online reviews of new products
*R_g_*_4_	Reputation revenue from the government’s regulatory measures
*C_g_*	Government’s supervision cost
*S*	Production subsidies or tax-free subsidies obtained by enterprises investing in research and development
*P*	Penalty imposed by the government on enterprises that have cheated or produced substandard products
*R_e_*_1_	Revenue from sales of new products by enterprises
*R_e_*_2_	Revenue from the sale of paper products by the enterprise
*R_e_*_3_	Company’s revenue from consumers’ participation in online reviews of new products
*C_e_*_1_	The investment cost of R&D products produced by the enterprise (*C*_*e*1_ > *C*_*e*2_)
*C_e_*_2_	Company’s cost of producing paper products
*U*_1_	Consumer’s utility of using new products (in direct proportion to the positive impact of online reviews of products on the company)
*U*_2_	Consumer’s utility of using paper products (in direct proportion to the positive impact of online reviews of paper products on the company)
*R_c_*	Consumer’s revenue from own participation in online reviews of new products
*L*	Consumer acceptance of new products
*u*_1_	Degree of positive influence of the online public opinion on a new product on the company (proportion of positive emotions in online reviews of the product)
*u*_2_	Degree of positive influence of the online public opinion on a paper product on the company (proportion of positive emotions in online reviews of the paper product)
*θ*	Probability of fraud
*β*	Probability that the government finds fraud companies
*γ*	Probability of consumers participating in online reviews
*α*	Threshold of *u*_1_
*l*_1_, *l*_2_	Threshold of *L* (*l*_1_ < *l*_2_)
*x*	Probability of government regulation
*y*	Probability of enterprise R&D (R&D: research and development)
*z*	Probability of consumer use of R&D products

**Table 5:** Government–enterprise revenue matrix.

Government	Enterprise	
	R&D (*y*)	No-R&D (R&D: Research and Development) (1 − *y*)
regulation (*x*)	*R_g_*_1_ + *R_g_*_4_ − *S* − *C_g_* *R_e_*_1_ + *S* − *C_e_*_1_	*R_g_*_2_ + *R_g_*_4_ − *C_g_* + *θ*[*βP* − (1 − *β*)*S*] *R_e_*_2_ − *C_e_*_2_ − *θ*[*βP* − (1 − *β*)*S*]
no-regulation (1 − *x*)	*R_g_*_1_ − *S* *R_e_*_1_ + *S − C_e_*_1_	*R_g_*_2_ − *θS* *R_e_*_2_ − *C_e_*_2_ + *θS*

**Table 6:** Equilibrium point and stable conditions of the game system between the government and enterprises.

Equilibrium Po int	*TrJ*	*DetJ*	Stable Condition	Results	Stability Strategy
(0,0)	−	+	*C_g_* − *R_g_*_4_ > 0; (*R_e_*_2_ − *C_e_*_2_) − (*R_e_*_1_ − *C_e_*_1_) − (1 − *θ*)*S* > 0	ESS_1_ (ESS: evolutionarily stability strategy)	no-regulation, no-R&D
(1,0)	−	+	*C_g_ − R_g_*_4_ + *θβ*(*P* + *S*) < 0 (*R_e_*_2_ *− C_e_*_2_) − (*R_e_*_1_ *− C_e_*_1_) − *θβ*(*P* + *S*) − (1 − *θ*)*S* > 0;	ESS_2_	regulation, no-R&D
(1,1)	−	+	*C_g_* − *R_g_*_4_ < 0; (*R_e_*_2_ *− C_e_*_2_) − (*R_e_*_1_ *− C_e_*_1_) − (1 − *θ*)*S* < 0	ESS_3_	regulation, R&D
(x*,y*)	0	0	−	Saddle point	−

**Table 7:** Government–enterprise–consumer revenue matrix.

Government	Enterprise	Consumers	
		UsingR&D Products (*z*)	Not Using R&D Products (1 − *z*)
Regulation (x)	R&D (*y*)	−	*R_g_*_1_ + *γR_g_*_3_ + *R_g_*_4_ − *S − C_g_* *L*[*R_e_*_1_ + *γ*(2*u*_1_ − 1)*R_e_*_3_ + *S − C_e_*_1_] *L*(*U*_1_ + *γR_c_*)
	No-R&D (1 − *y*)	*R_g_*_2_ + *γR_g_*_3_ + *R_g_*_4_ *− C_g_* + *θ*[*βP −* (1 *−* *β*)*S*] (1 − *L*)[*R_e_*_2_ + *γ*(2*u_2_* − 1)*R_e_*_3_ *− C_e_*_2_ − *θ*[*βP −* (1 *−* *β*)*S*]] (1 − *L*)(*U*_2_ + *γR_c_*)	*R_g_*_2_ + *γR_g_*_3_ + *R_g_*_4_ − *C_g_* + *θ*[*βP −* (1 *−* *β*)*S*] *R_e_*_2_ + *γ*(2*u_2_* − 1)*R_e_*_3_ *− C_e_*_2_ − *θ*[*βP −* (1 *−* *β*)*S*] *U*_2_ + *γR_c_*
No-regulation (1−x)	R&D (*y*)	*R_g_*_1_ + *γR_g_*_3_ − *S* *R_e_*_1_ + *γ*(2*u*_1_ − 1)*R_e_*_3_ + *S − C*_e1_ *U*_1_ + *γR_c_*	*R_g_*_1_ + *γR_g_*_3_ − *S* *L*[*R_e_*_1_ + *γ*(2*u*_1_ − 1)*R_e_*_3_ + *S − C_e_*_1_] *L*(*U*_1_ + *γR_c_*)
	No-R&D (1 − *y*)	*R_g_*_2_ + *γR_g_*_3_ − *θS* (1 − *L*)[*R_e_*_2_ + *γ*(2*u_2_* − 1)*R_e_*_3_ *− C_e_*_2_ + *θS*] (1 − *L*)(*U*_2_ + *γR_c_*)	*R_g_*_2_ + *γR_g_*_3_ − *θS* *R_e_*_2_ + *γ*(2*u_2_* − 1)*R_e_*_3_ *− C_e_*_2_ + *θS* *U*_2_ + *γR_c_*

**Table 8:** Equilibrium point and eigenvalues of the Jacobian matrix corresponding to each equilibrium point.

Equilibrium Point	Eigenvalues		
(0,0,0)	*R_g_*_4_ + *θβ*(*P + S*) − *C_g_*	*LQ*_1_ − *Q*_2_	− *L*(U_2_ + *γR_c_*)
(1,0,0)	*C_g_* − *R_g_*_4_ − *θβ*(*P + S*)	*LQ*_1_ − *Q*_2_ + *θβ*(*P + S*)	− *L*(U_2_ + *γR_c_*)
(0,0,1)	*R_g_*_4_ + *θβ*(*P + S*) − *C_g_*	*Q*_1_ − (1 − *L*)*Q*_2_	*L*(U_2_ + *γR_c_*)
(1,1,0)	*C_g_ − R_g_*_4_	*Q*_2_ − *LQ*_1_ − *θβ*(*P + S*)	(1 − *L*)(U_1_ + *γR_c_*)
(1,0,1)	*C_g_* − *R_g_*_4_ − *θβ*(*P + S*)	*Q*_1_ − *Q*_2_ + *θβ*(1 − *L*)(*P + S*)	*L*(U_2_ + *γR_c_*)
(1,1,1)	*C_g_ − R_g_*_4_	*Q*_2_-*Q*_1_ − *θβ*(1 − *L*)(*P + S*)	(1 − *L*)(U_1_ + *γR_c_*)

## Data Availability

The data used to support the findings of this study are available from the corresponding author upon request.
